# “*Warmi urquy”*: marriage proposals and gender hierarchy in the Peruvian Andes

**DOI:** 10.3389/fsoc.2026.1722698

**Published:** 2026-03-03

**Authors:** Edgar Gutiérrez-Gómez, Luis Miguel Flores-Vargas, Rosa Cecilia González-Ríos, Sonia Beatriz Munaris-Parco, Jaime Carmelo Aspur-Barrientos, Aldo Bazán-Ramírez

**Affiliations:** 1 Facultad de Ingeniería y Gestión, Escuela de Administración de Turismo Sostenible y Hotelería, Universidad Nacional Autónoma de Huanta, Ayacucho, Peru; 2 Área de Ciencias Sociales, Colegio Nacional “Nuestra Señora de Fátima”, Ayacucho, Peru; 3 Departamento de Educación y Humanidades, Universidad Nacional José María Arguedas, Andahuaylas, Peru

**Keywords:** Andean culture, arranged marriage, gender, Quechua culture, sexism

## Abstract

The warmi urquy ritual, still practiced in the province of Huanta, Ayacucho region, is a tradition of marriage proposal in the Peruvian Andes, in which gender hierarchies and sexist social norms are evident. The objective of this research is to understand the cultural and social significance of this practice by analyzing the manifestation of gender roles and power relations during the ceremony. The research is conducted using a qualitative approach with ethnographic methodology, combining participant observation, semi-structured interviews, and informal conversations. Direct participation in the ceremony allowed for the recording of the roles of the actors, symbolic expressions, and perceptions of godparents, family members, and guests. The results show that women are at the center of the ritual, while the groom and his family seek to demonstrate their performance and ability to “take her out” as a wife, evidencing social control and gender hierarchy. In conclusion, this study demonstrates the persistence of Andean traditions despite social and cultural changes.

## 1 Introduction

Andean communities in Peru are characterized by their unique forms of social organization and coexistence, in which their self-governing nature reduces the effectiveness of national laws. For centuries, the marriage system has been markedly sexist. The marriage proposal, known in Quechua as warmi urquy, consists of taking the woman from her home through rituals specific to each community. In this context, “Quechua culture has its own idiosyncrasies, represented especially by the current Quechua-speaking Andean communities. This Quechua society has specific forms of social coexistence” (Gutiérrez-Gómez et al., 2024, p. 8). This shows how cultural traits are expressed in community life and in social practices that preserve their identity.

Recent social mobility has intensified, especially from rural areas to cities, transferring customs and traditions that remain alive today. Forced displacement due to social phenomena has made this dynamic evident. In this regard, “Isabel Coral, one of the leading experts on displacement in Peru, proposed the figure of 600,000 displaced persons in the 1990s, which later became popular among specialists; her current position on the issue is very critical” (Ramírez Zapata, 2018, p. 680). This quote shows how Coral influenced the estimation of displaced persons in Peru, although today she critically questions those published figures. It is important to note that the Peruvian highlands and their Andean communities were the most affected by these displacements. Likewise, in this context of interculturality and the prevalence of social practices, it is noteworthy that “paternalistic practices are deeply rooted in the political and social structures of the province of Huanta” (Gustafsson, 2010, p. 145). Paternalistic practices persist in Huanta, profoundly influencing the political and social organization of the province.

The study was conducted in the province of Huanta, where migrants still practice marriage proposal rituals through special ceremonies typical of their villages. This practice is a way of keeping their traditions alive in a cultural context where “the ancient Andean peoples conceived of sexual acts and sexual relations between men, women, animals, deities, and the dead” (Hill, 2015, p. 1). The Andean worldview gave sexuality a transcendent character that integrated nature, spirituality, and sacred beings into various practices. There are surprising stories about the process of asking for a woman’s hand in marriage, which include suffering, rejection, heartbreak, envy, and revenge between the families of the bride and groom. Although Huanta is now considered an urban province, a large part of its population comes from Andean communities that have been displaced or mobilized by various factors, and it is they who preserve these ancestral practices of warmi urquy, often imbued with macho rituals.

The tradition commonly known as asking for someone’s hand in marriage is a practice with Roman and medieval roots. In the Quechua language, it is called Warmi Urquy and takes on particular nuances depending on each Andean culture or community. In the Andean context, colonial influence has been incorporated into this ritual: “Asking for someone’s hand took on a more romantic aspect by also including engagement rings and kneeling to propose marriage. Precisely this last custom, which dates back to the Middle Ages, reversed the aspect of women’s submission to men” (Sánchez, 2022, para. 2). This shows a symbolic transformation, which went from patriarchal control to a romantic and chivalrous gesture, although traditional structures still persist in marriage commitments. In the matchmaking process, couples usually know each other beforehand. According to the testimonies collected, in the past this bond was established without any prior knowledge between the future spouses. Nowadays, the marriage proposal is carried out as a ritual that seeks to consolidate respect between families and preserve tradition.

Another determining factor is the presence of godparents, who play a significant role both in the ceremony and in the preparatory stages. Despite social heterogeneity and the advance of modernity, these customs remain unchanged, as “the province of Huanta is located in the Ayacucho region, in the central Andes of Peru. It is divided into seven districts distributed across three areas: the valley, the highlands, and the tropical rainforest” (Gustafsson, 2010, p. 144). Thus, it can be observed that Huanta maintains ancestral practices that influence the formation of new families, where divorce is not a constant, unlike in contemporary urban contexts.

## 2 Methodology

This is a qualitative research study whose purpose is to understand the cultural and social significance of the warmi urquy ritual in the province of Huanta, Ayacucho region, Peru. The methodology used is ethnographic, which has allowed us to record, interpret, and analyze the symbolic aspects, ritual practices, and perceptions associated with this traditional marriage proposal. The fieldwork combined participant observation, semi-structured interviews, and, especially, informal conversations. Participant observation was carried out during the celebration of the warmi urquy ritual (“taking the woman from her home”), which allowed for the recording of the most relevant roles of the participants and the interaction between the different social actors.

Participant observation allowed us to collect symbolic expressions, implicit discourses, and meaningful gestures that would be difficult to capture using other instruments. Interviews were conducted with older people, the groom’s parents, relatives, godparents, and ceremony attendees, which allowed us to identify the diversity of perceptions about the practice of the ritual, particularly in relation to its hierarchical and sexist nature. The researchers are fluent in Quechua, which facilitated interaction and understanding of the most essential elements of the ritual. The research was conducted in the city of Huanta, which has a population of approximately 40,000. Participants were selected intentionally, based on family ties; therefore, the researchers became part of the groom’s family, which limited their direct access to the bride’s family.

There was also a limitation in terms of interaction with the bride’s family and the bride and groom, who were busy during the ceremony. There are various accounts of the bride’s families that could not be addressed in this research. The Quechua language barrier was another challenge, as literal translation sometimes alters the meaning of expressions; however, an effort was made to maintain the essence of the messages in the interpretation. Field notes, interview transcripts, and observation records were subjected to thematic analysis, which allowed for the identification and categorization of recurring patterns related to the symbolism of the ritual, its forms of social control, and gender hierarchies.

## 3 Results

### 3.1 Naturalization of women’s subordinate role in the *“warmi urquy”* ritual

In rural communities, wives are seen as the backbone of the household, mainly responsible for cooking and domestic work. This view is reflected in the warmi uruy (marriage proposal), which is intended to lead to marriage and requires visiting the bride’s home at least three times. The first visit, called kichkachi (making the groom’s good intentions known), is characterized by the fact that, in most cases, the families and the couple do not know each other and have not had any previous contact. Under these practices, “women are represented in a state of subordination, thus reinforcing traditional gender roles and perpetuating established power hierarchies” (Zhang, 2024, p. 60). Participant observation also reveals that: “women must have demonstrable knowledge of domestic chores, they are instructed to wash, cook, serve their husbands, behave well, be at their husbands’ command, raise children, and follow a series of advice given by all family members.” These practices reveal a cultural pattern that assigns women a subordinate role, restricted to domestic tasks and family obedience.

The narrative surrounding Andean marriage, especially in the province of Huanta, is characterized by traditional practices developed in an environment with limited access to information. Elderly women and men interviewed point out how difficult it is to accept an unknown husband, a situation that is commonly facilitated by the intervention of the godfather in collusion with the father of the bride and groom. In this context, it is understood that “a daughter is one of a family’s most precious treasures and cannot marry just any man who will not respect and love her” (Sánchez, 2022, para. 12). The Andean marriage proposal, known as warmi uruy, takes place in private ceremonies attended only by close family members, which forced researchers to present themselves as relatives of the man in order to gain access to the ritual. The gathering at the groom’s house includes the arrival of each family member with a bottle of liquor and the indispensable coca leaf, elements that play a symbolic and functional role in keeping the attendees awake.

**Figure 1:**
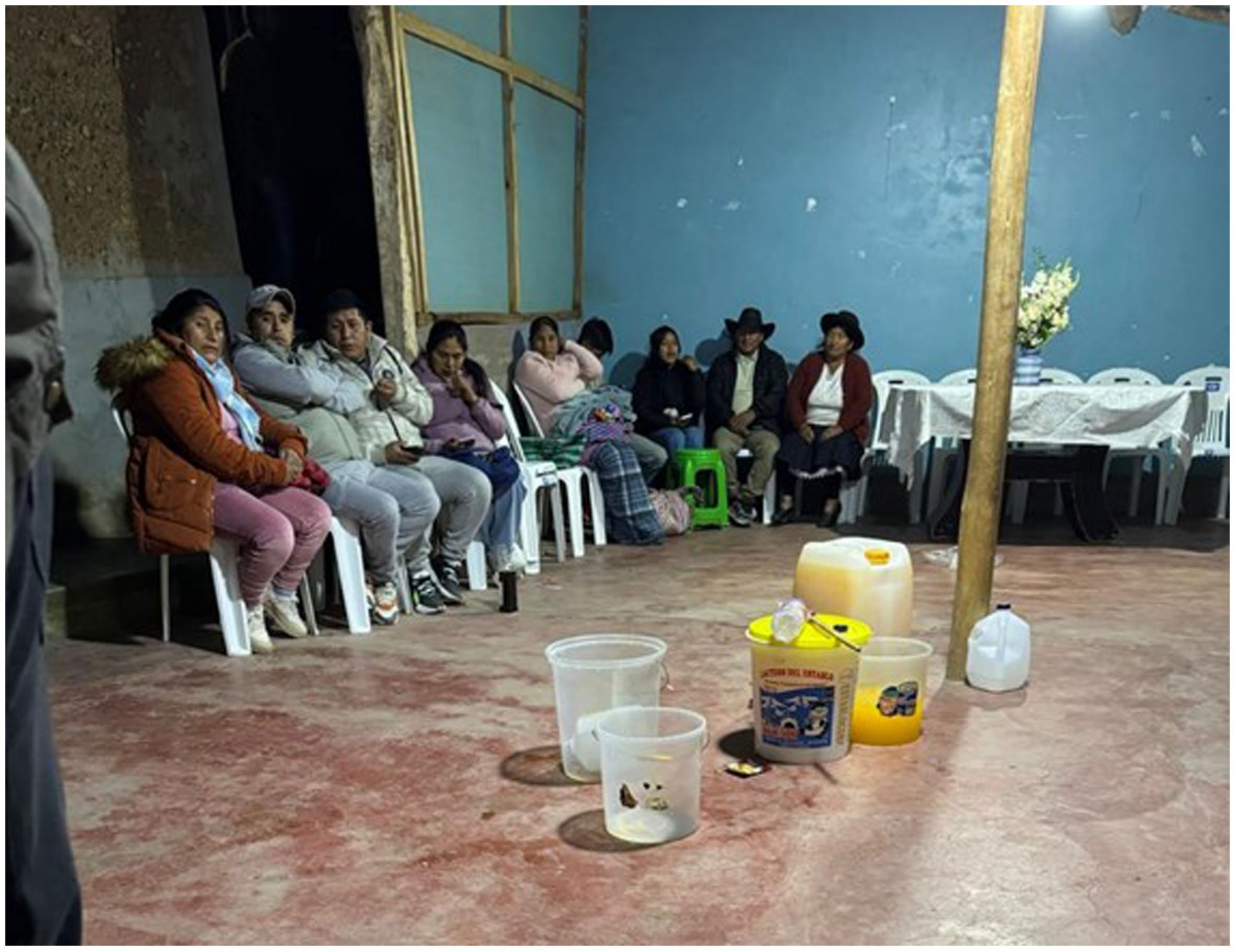
At the groom’s house, prior to the ceremony, the male guests gather to prepare drinks and food, including the traditional mondongo (based on made from corn and sheep offal) intended for the following day. Datum: image recorded by researchers, June 2025.

The warmi urquy ritual is usually performed at night. One interviewee states: “It is done at night because it is associated with stealing a chicken (which is the woman), and there is also a saying that Ayacucho men are warmi gusto (they like women) until death.” This perception reflects an Andean worldview in which marriage symbolizes the appropriation of women, reinforcing gender stereotypes and male dominance. The progressive shift toward gender equality is not evident in the Andean marriage proposal ritual, as “the oppression of women is exacerbated by the traditions, cultural beliefs, and religions of most societies, which favor patriarchy. Women do not dare to challenge the status quo” (Nyanta et al., 2017, p. 14). This shows how traditions, beliefs, and religions reinforce patriarchy, limiting women and preventing them from questioning oppressive structures. Another interviewee adds: “It is essential to comply with these ritual mandates because it guarantees the duration of the marriage; look at me, I am always with my wife.” This refers to his experience of a ritual when he was young. Figure 1 shows the groom’s house at the beginning of the night ceremony.

Relatives often ask themselves several times whether the groom, his parents, and the best man have completed the first stages of joining the bride’s family. The process begins with the *kichkachi* (a preliminary meeting without knowing each other), continues with the second entrance (agreements), initials on how the ritual should be carried out and culminates in the third, when everything has been agreed upon and the woman is taken to the man’s house. In the Andean communities of Peru, this practice is accompanied by social self-management and moral censorship, unlike what happened during industrialization, where “the new gender system introduced in the new industrial world of factories was based on the principle of female inequality and subordination, and was backed by law” (Nash, 2008, p. 75). While industrialization institutionalized gender inequalities through laws, in the Andes the ritual functions as a mechanism of social protection, given that women are considered vulnerable to men, and any abuse forces the family to intervene by virtue of the commitments made during the ceremony.

At the groom’s house, preparations for the ritual seek to make a favorable impression on the bride’s family. Participant observation shows that the main concern is to find out how attached the bride’s family is to tradition. In this context, stories and anecdotes are shared about previous experiences of marriage proposals in the villages of origin. Comments arise linked to prevailing machismo, questioning the continuation of a ritual focused solely on women and raising the need for greater equality. Likewise, the participants take great care with certain symbolic elements: checking the coca leaf, preparing the food with care, and ensuring that the drinks are served in plastic containers, as glass ones, which break easily, are considered to bring bad luck. This set of practices and perceptions allows us to understand that “the relationships of subordination between men and women who are married and live in households should not be taken to mean that these are the only social relationships in which gender is significant” (Whitehead, 1979, p. 11). In this way, it is emphasized that gender dynamics transcend marriage and manifest themselves in various social interactions.

### 3.2 Symbolic affirmation of machismo in Andean marriage practices

The ritual focus is on the female figure: what she will be like, her family, and whether the offering rituals will be to her liking. The groom’s mother shows greater distress. At stake is not only his son, but also the image of the entire family. Participant observation reflects: “The entire ritual takes place throughout the night, beginning days in advance with the hiring of musicians, godparents, roast pig, *qarawi* (singing in Quechua, performed only by women), especially elderly women, who prepare the mondongo, chicken broth, food service, and beverages.” This situation resembles “a reputation that for a long time has turned its inhabitants into ‘machos among machos,’ [men’s meeting] since, historically, nationalism and masculinity are intertwined. A history marked by struggles around different representations and imaginaries of nationality” (Machillot, 2013, p. 167). This reflects how historical nationalism confused virility and homeland, consolidating strong masculine identities linked to struggles and collective imaginaries.

Ancestral knowledge, recognized and practiced by various communities, has survived with variations depending on each family and town. Even so, the sexist burden that historically relegated women to a secondary role linked to domestic tasks is not always identified: “it is possible to affirm that expressions of sexist violence, far from disappearing quickly, have become established in ways that are more difficult to detect and question” (Redondo, 2019, p. 128). One interviewee argues: “It is a long-standing tradition to practice these rituals, and this is how the family endures over time, because nowadays there are constant divorces. It indicates that the elders never divorce and maintain family unity. In his case, it was the same with the marriage proposal (warmi urquy), where they had to go far from their village and thus remain together up to the present.” This testimony it shows how marriage proposals operate as a cultural mechanism that reinforces marital stability, contrasting the continuity of tradition with contemporary marital instability.

**Figure 2:**
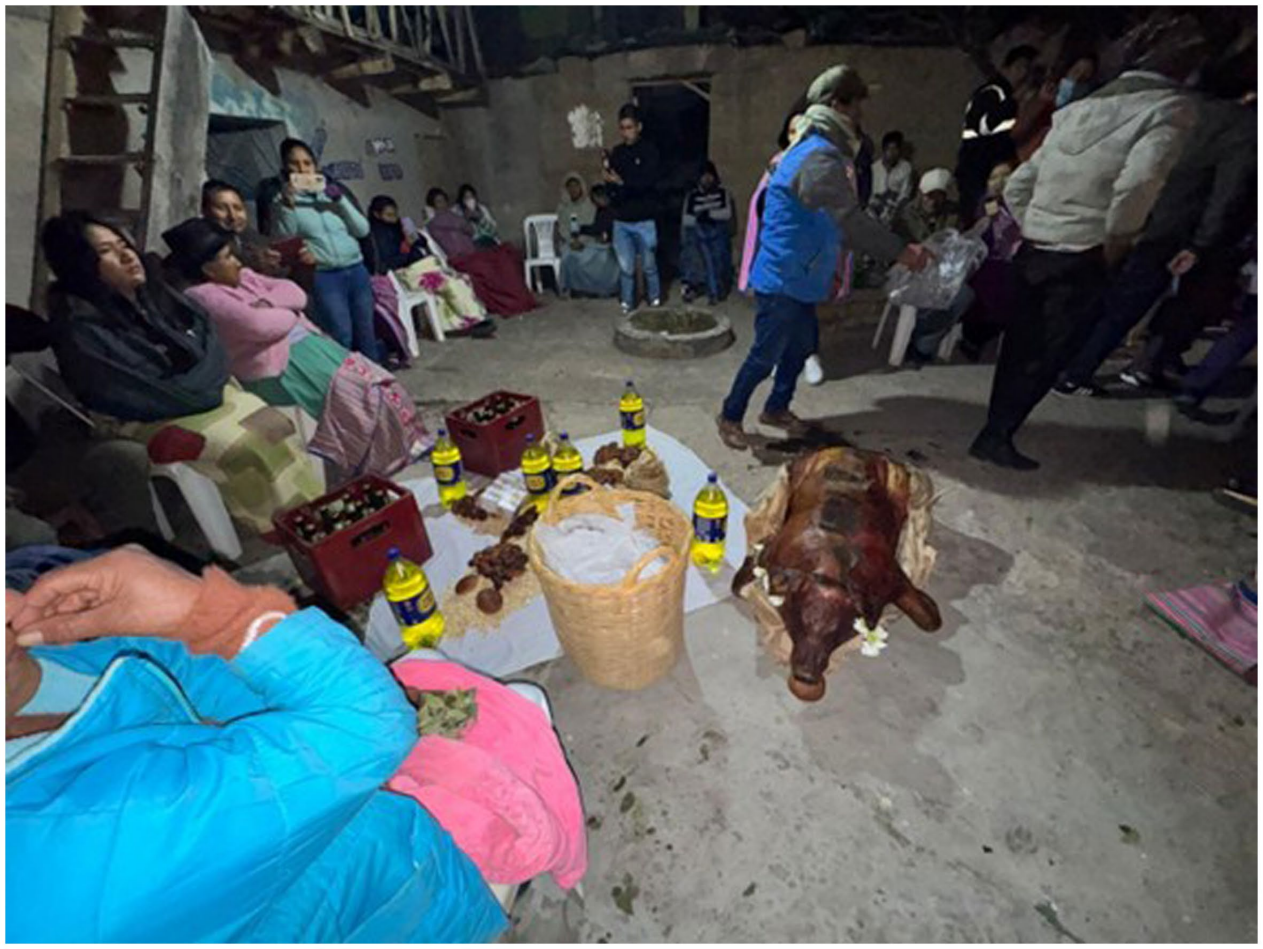
The products on display symbolize the change of the bride’s daughter take it to the father’s house. Usually, roasted corn, breads, soft drinks, two cases of beer, and, as an essential element in every warmi urquy, the roast pork. Datum: image recorded by researchers, June 2025.

The practice of symbolically giving away products as part of ancestral tradition reveals a hidden form of machismo: it is up to the man’s family to organize and pay for the preparations with the aim of impressing the woman’s family. The field note recorded: “The conversation revolves around the different anecdotes they had about the rituals involved in proposing marriage. Some say they suffered greatly in finding a wife because their parents were very demanding in terms of fulfilling certain rituals. The younger men are excited to be part of the warmi urquy for the first time.” This dynamic reveals how traditional experiences reproduce gender inequalities. As points out: “It is difficult to separate men’s sexist acts from the effects they have on women. Therefore, women can play a fundamental role in explaining machismo” (McGuire, 2013, p. 50). This highlights the leading role of women in the critical understanding of machismo. Figure 2 shows female subordination in the delivery of the roast pig, a tradition deeply rooted in this type of ritual and constantly demanded by the bride’s family.

The testimonies gathered in the conversation indicate that marriages used to be almost arranged, with godparents playing a leading role and then treating the newlyweds as children at their disposal. In this sense, “arranged marriages have declined in prosperous countries with greater social mobility and growing individualism, as there is a negative perception that these marriages are entered into solely to satisfy the needs of the parents” (Myrlinda, 2019, p. 141). It is observed: “The commissioner places the blanket and white tablecloth in front of the bride’s parents and decides to spread corn toast (cancha, qamka) in the shape of a cross, while at the same time asking another commission to bring the other supplies such as: wawa bread, Inka Cola soda, a couple of bottles of cane liquor, two boxes of beer, packaged rolls, and finally roast pork.” This reflects how material symbols and ritual foods strengthen family ties, highlighting the spiritual and communal dimension of the proposal. Each element has a meaning associated with well-being: products of the earth, such as corn; bread, an essential food that must always be present; and soda, considered the best Peruvian brand and arranged in the shape of a cross as a protective symbol.

Warmi urquy is quite popular in the Peruvian highlands, where there are reports of extreme machismo that seem to surpass fiction. These practices include divine references among religious congregations, with frequent allusions to the Bible especially the story of Adam and Eve. As noted, “this strategy not only fosters attractive environments for religious debates, but also cultivates online religious communities, transforming traditional messages to adapt them” (Gutiérrez-Gómez et al., 2025, p. 613). Participant observation indicates: “The person responsible for knocking on the door and conducting the ritual ceremony peeks out suspiciously to speak to the bride’s parents, who have arrived with the good intention of asking for their daughter’s hand in marriage. At that moment, the grandfather intervenes and suggests that the father should speak. Then, the father, quite fearful, takes the floor, recalling that every family had to go through this stage, and refers to the Bible, mentioning the story of Adam and Eve, to begin his speech in Quechua.” This shows how the father, with fear and family support, legitimizes the ritual by appealing to both the Bible and Quechua tradition.

### 3.3 Ritualization of family consent as a mechanism of control over women

Some testimonies from those who experienced Warmi urquy and participated in the ritual are not positive. They argue that this tradition should be modified, as they consider it very old-fashioned and sexist, as in the following case: “Her desire was to continue in school, but her family situation prevented her from doing so. She recalls that ‘my dad scolded me when I wanted to read because he said it was to write letters to my boyfriend. Women could not write or read’” (Uzeta Iturbide, 2004, p. 8). This experience contrasts with what is observed in ritual practice: “The father of the groom, together with the godparents, organizes a delegation with specific functions. One of these is to designate the person responsible for knocking on the door of the bride’s house, who must be trained in advance in what to say, as most of the dialogue is in Quechua. This task falls to someone with experience and good fortune in previous marriage proposals. The person in charge repeats phrases such as qamunico sumaq rusas waitachayquiman (we come to your beautiful virgin flower), as the woman is compared to a flower. It is essential to pronounce the word rusas (virgin), as omitting it can be interpreted as an offense. When the woman already has children, the questions must be phrased differently. In such cases, there have been instances where the bride’s father has refused to open the door”. The procession, led by the father and godparents, chooses an expert spokesperson who recites ritual phrases of respect in Quechua.

The gathering of family members, particularly the older ones, is a central element in the warmi urquy, as they are the ones who endorse the seriousness of the ritual. According to “the experience of love is part of the human experience, and what is experienced during this process seems to be so powerful that it brings humans closer to the divine” (Cirineu, 2020, p. 4). This spiritual nature contrasts with the practical dimension described in the interview with the door commissioner: “What happens if they don’t open? He says it’s a tradition to open before midnight, because otherwise it’s bad luck. Besides, it has already been agreed that this is Warmi Urquy’s third visit, they are a beloved couple, and there would be no major inconvenience”. Thus, the tradition of opening the door before midnight is understood as a symbol of good luck, while the prior agreement confirms the acceptance of the union.

The request for a man’s hand in marriage (qari urquy) does not exist in Andean communities, as corroborated by the testimonies collected. In this way, ritualization becomes a Family control mechanism against the risks of divorce: in the event of marital conflicts, godparents are the first line of support. Consequently, they must have a solid family background and be respected members of the community. During the ceremony, the godparents publicly affirmed that they were acquiring their ninth child, expressing satisfaction and pride in having more godchildren. This exercise of social control is particularly relevant in some Andean communities, where it is evident in “the symbol of social control of the Varayoc woman, who broke the male stereotype that had persisted since the time of the ancient Incas” (Gutiérrez-Gómez et al., 2023, p. 7). According to tradition, the staff represents the male organ, and in ancient times only men were allowed to carry it. Today, women have gained access to positions of authority and also carry the staff of office in various Andean communities. In the same context, the following observation was made: “This is how the task of knocking on the door begins, starting with the groom’s father, who it is a very complicated job that involves a lot of nerves, because it is an unknown family and they must respect their way of life, and they do not know how they will react”. However, these practices show variations and changes in other sectors of the Andean community.

**Figure 3:**
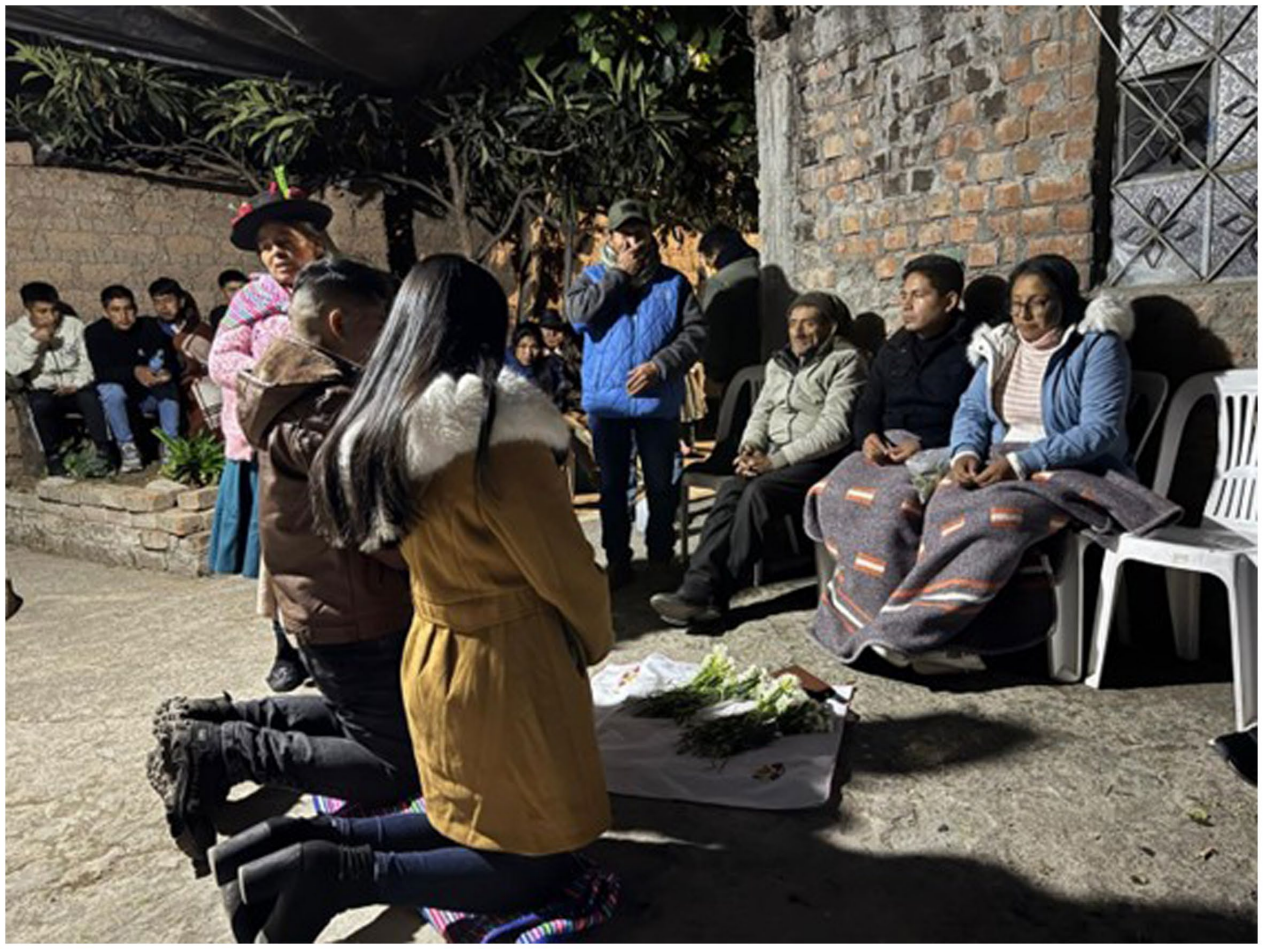
The bride and groom are kneeling for over an hour (2:30 a.m.), listening to the moral sermons of their closest relatives, who approach one by one. Some attendees ask that he be break with ancient tradition. Datum: image recorded by researchers, June 2025.

In the ritual scene, the main focus is on the bride. Both the groom’s family and the bride’s family observe her clothing, age, beauty, and bearing with anticipation. Some attendees stated that in other communities, brides still wear traditional clothing; however, in this case, a more modern aesthetic was chosen, which was questioned by the older adults. In this ceremony, there is no evidence that “Andean women fulfill functions oriented toward the sense of duality known as panipacha. This interaction implies the sense of solidarity, reciprocity, and equality between men and women” (Mamani, 2010, p. 91). The quote highlights the panipacha duality, where men and women interact in reciprocity and solidarity, ensuring complementary equality within the community. On the contrary, observation reveals the family’s dominance over the bride and groom, expressed in another more demanding ritual: “another more important and complicated ritual begins, the presenter asks for a blanket to be brought and placed behind the white tablecloth with flowers, where the bride and groom must kneel to receive sermons from almost all of the bride’s and groom’s relatives, starting with the bride’s relatives.” This is illustrated in Figure 3.

**Figure 4:**
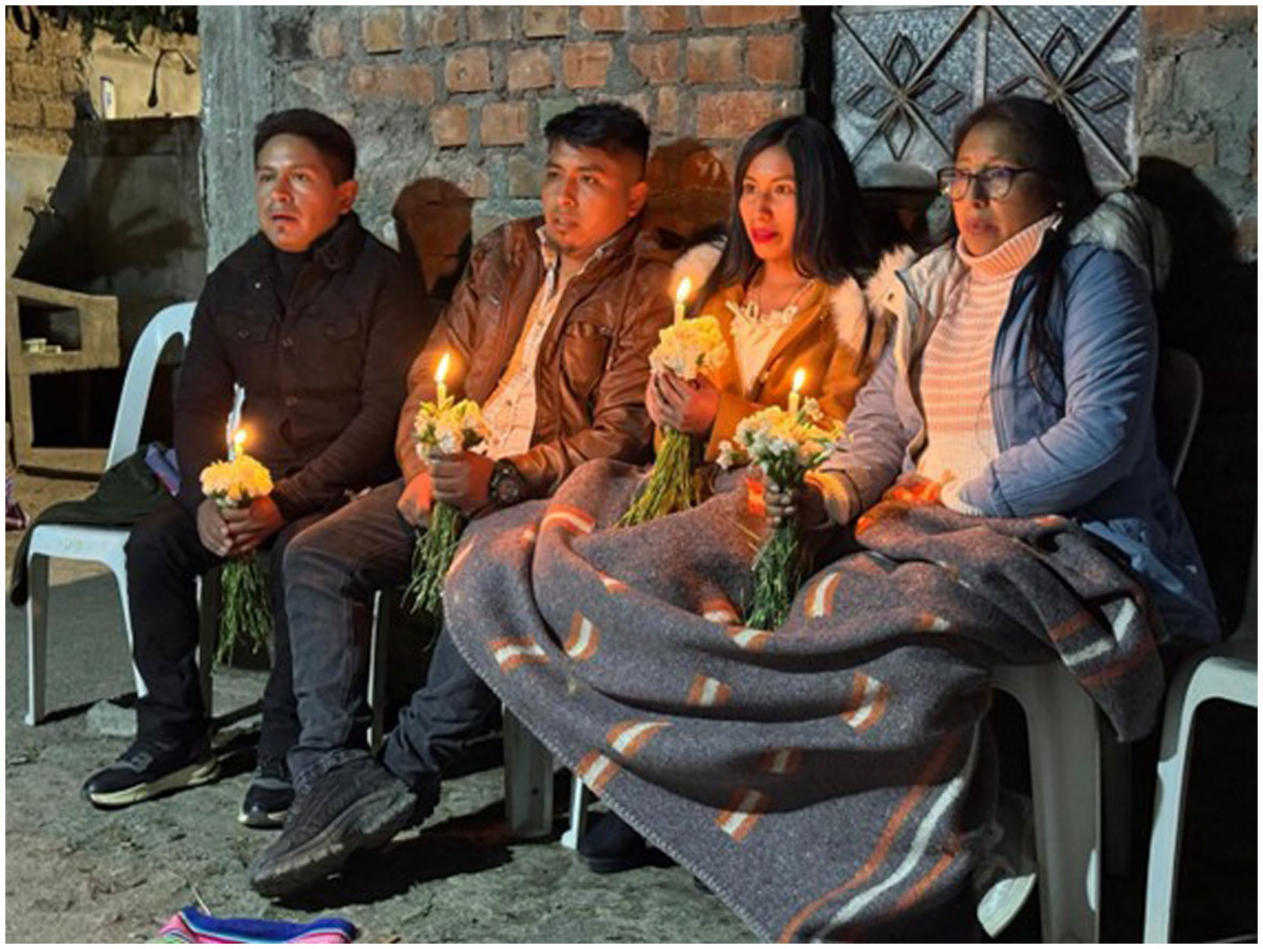
After the sermon ritual has been completed, the bride and groom are handed over to the godfather. They remain with the candle lit, which must not be extinguished, as this would be a bad omen. Datum: image recorded by researchers, June 2025.

Andean customs cannot be understood as uniform, since Peru, being a multicultural country, shows variations in its rituals from one community to another and even between families. This diversity breaks with the assertion that “in the Andean world, women were responsible for ceremonies dedicated to female deities, while men were in charge of rituals related to male gods” (Rodríguez García and Mó Romero, 1998, p. 148). Ethnographic observation reveals the contradiction: “Among the many pieces of advice, women are reminded that they must know how to cook, wash clothes, look after their children, respect their husbands, and help with the godparents” chores; in essence, these are recommendations aimed at harmonious coexistence with a marked emphasis on domestic work. Similarly, men are told that, from now, he must leave his friends behind, abandon his bachelor lifestyle, and, above all, give up his cell phone, considered a device that separates families. He is also urged not to dishonor his family with bad behavior, remembering finally that children are symbolically handed over to their godfather (Figure 4).

The family control mechanism is strongly evident in the new couple, who begin a process in which they must internalize the sacrifice made in extreme conditions, at 2 °C below zero. The visit by the man’s family, made out of consideration for the bride and at the request of her family, was repeatedly highlighted. The participant observation recorded: “A once the sermon by the parents and close relatives has concluded, another stage begins, led by the godparents, who carry a pair of rosaries and perform what is known as the exchange of rosaries”. Each places the rosary around the other’s neck and then performs a symbolic exchange accompanied by prayers. At this point, the bride and groom are asked to stand up and light the candle together, along with the flower placed on the white blanket throughout the ceremony. The flame must not be extinguished, as this is considered a bad omen. There has been a slight improvement compared to accounts of older rituals, although it is confirmed that “in the past, only men could go to school. Our parents considered that it was not necessary for women to know how to read and write, that it was enough to know how to herd pigs and sheep and how to cook” (Rodríguez García and Mó Romero, 1998, p. 35). The quote highlights the historical educational inequality, where men were privileged and women were relegated to domestic and pastoral tasks.

### 3.4 Contemporary reinterpretations of ritual by young Andean women

There is tension between maintaining tradition and questioning its permanence in a context with macho connotations, such as the warmi urquy. In the interview, the couple argue that it is the tradition of their parents and their people, and therefore must be preserved. However, this reinterpretation should not be separated from the fact that “indigenous women added to their status as Indians the subordination due to gender that catapulted them into a more invisible and secondary position” (Rodríguez García and Mó Romero, 1998, p. 154). This statement denounces a double oppression: ethnicity and female identity, placing indigenous women in a socially, historically, and structurally secondary invisibility. Participant observation reaffirms: “Once the rosary has been exchanged and the symbolic engagement ring has been given to the bride, they sit next to their godparents and another stage of the ritual begins. The person in charge of knocking on the door asks the materials committee to bring a blanket and a white tablecloth, and spreads it out in front of the bride’s parents.” The godparents are relatively young people, unlike their companions.

**Figure 5:**
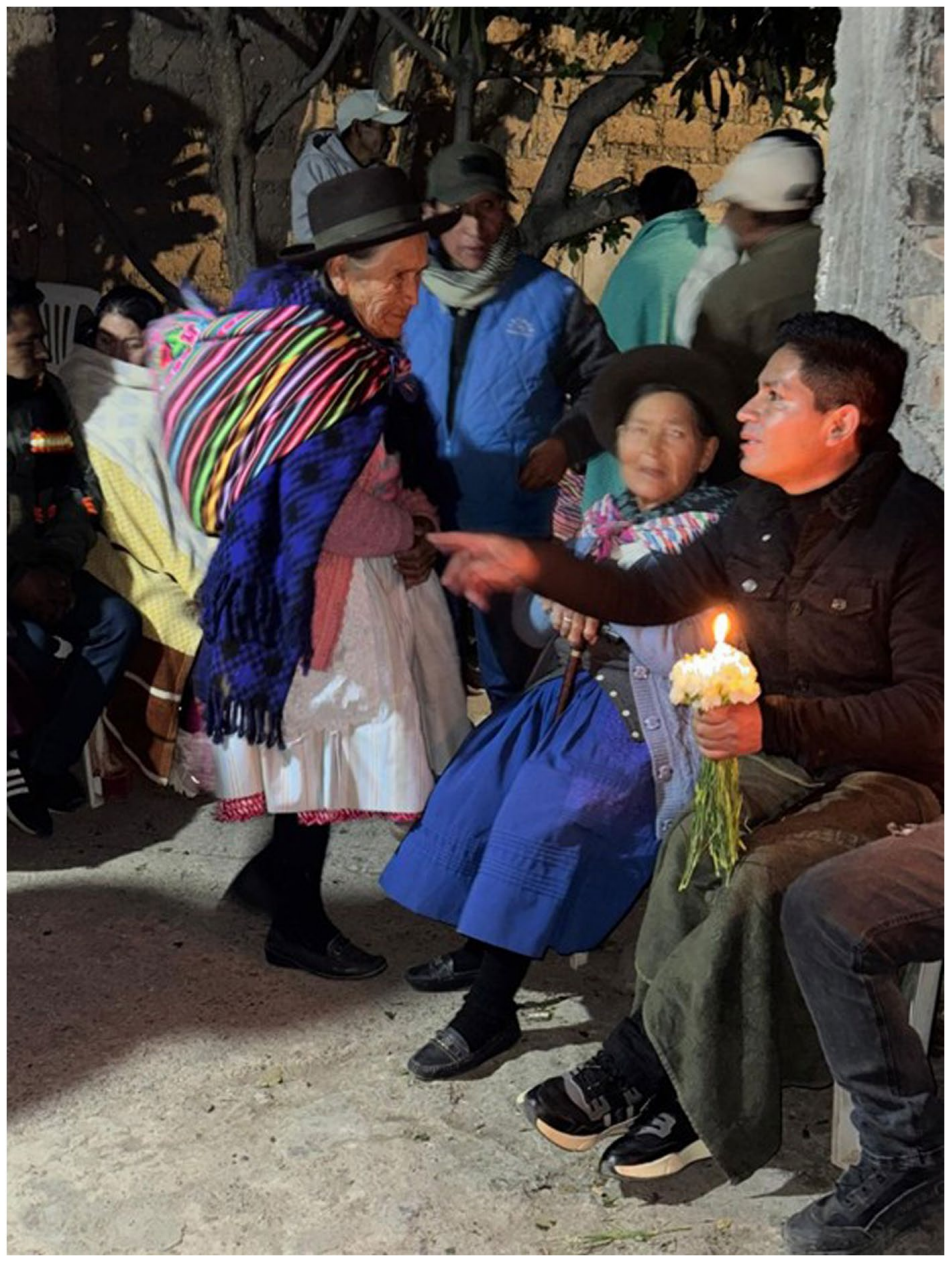
The two elderly women who sang “qarawi,” a traditional song requesting a marriage proposal, rebuke the godfather for not performing the traditional rituals with more enthusiasm. They will be the guardians of tradition. Datum: image recorded by researchers, June 2025.

Family sermons emphasize the need to start a family with children, assigning women the responsibility of raising them, which is framed as a “misunderstanding sociocultural norms rooted in racism and sexism toward rural indigenous women. This broader context, I suggest, explains why it was possible for medical personnel to resort to coercion in family planning programs” (Boesten, 2007, p. 10). During participant observation: “It was already two in the morning, the cold was intensifying, and, at the warmth of the exchange created an atmosphere of trust with the words of each attendee. At that moment, the grandfather intervened and reminded everyone of something important: before collecting all the offerings, the wedding date had to be set”. Older people show a strong attachment to tradition, and their sermons are often laden with male-dominated discourse, in which the godparents are addressed, as can be seen in Figure 5.

Once the sermons, rosary changes, delivery of the roast pig dowry, and other formal rituals in Quechua have been completed, permission is requested for the presence of musicians, a request that is granted by the bride’s family. Observation: “At that moment, the groom’s father asks to speak to ask if the musicians can come in, and this is authorized. Thus, the party begins with harp and violin music; they begin to drink liquor and all the families start dancing.” These ritual moments are very important, as they constitute serious instances of cultural affirmation, since they allow “open participation in the political and, above all, religious activities of the community, they were now able, from this less visible position, to maintain and even promote traditional customs and rites” (Rodríguez García and Mó Romero, 1998, p. 155). This highlights how the actors social practices, even from less visible places, manage to preserve and reinforce the collective practices of the community.

Once the drinking and dancing rituals were over, at around four in the morning, something unexpected happened. The participant observer describes: “At around four in the morning, something unusual began to happen: it was time to leave and take the bride away. However, her family decided to continue drinking, as they believed that this was the only way to express their affection. The celebration continued with dancing and drinking. The surprise was that the commissioner on duty was completely drunk, as everyone had toasted with him.” The bride’s departure from her home was hurried, resembling an escape; in this context, the groom’s family began a demonstration of achievement, taking the bride away as a symbol of triumph. Around five in the morning, the procession boarded the vehicle that waited outside to move to the groom’s house, where the real party begins as the culmination of the day. These types of dynamics must be analyzed considering that “men supported the movement by carrying signs that read: ‘beware, machismo kills.’ In the indigenous region of Puno, Peru, Aymara women marched with banners that embodied their relationship with Pachamama, an Andean deity” (Cavagnaro and Shenton, 2019, p. 14). This reflects how urban and Andean identities articulate different forms of resistance: men against machismo and Aymara women in relation to Pachamama.

The party continued all day at the groom’s house; none of the woman’s relatives accompanied her in what was considered an elopement with the “trophy,” which was the bride. Another person was waiting at the house. A group of people was in charge of preparing tripe and chicken soup for the relatives who had spent the night awake. According to estimates by those close to the family, the total cost of the celebration averaged five thousand dollars. Others pointed out that it is an excessive expense that creates commitments for both families and communities. Likewise, there are testimonies circulating that are laden with irony and a strong dose of machismo, questioning women’s honor and physical appearance. In Andean society, as well as in formal education centers, dances and performances such as the “warmi urquy” or the “yaukupakuy” (accompanying the person who is going to take the bride away, of a retributive nature).

## 4 Conclusion

The struggle for gender equality in Peru and other parts of the world is ongoing; however, Andean society has developed its own ways of preserving family unity in the absence of legislation that adequately responds to the needs of their communities. According to the interviews, it can be inferred that the practice of warmi urquy, as a traditional element, aims to protect women from possible situations of abuse by men. Given this vulnerability, the presence of both families during the ritual acts as a guarantee and safeguard for the union. The families and individuals who participate in this rite play a symbolic social control role, especially the godfather, who welcomes the new family as his own child and, consequently, assumes the responsibility for ensuring their good behavior and harmonious coexistence. In turn, godparents must be people of impeccable character in the community and constitute a model family in order to have godchildren at the engagement ceremony.

The study conducted in the province of Huanta shows that, although warmi urquy remains an ancestral practice, it also reproduces hierarchical and sexist structures that have historically relegated women’s autonomy. This ritual, which focuses on the suitor’s ability to “take” the woman from her home, reinforces the idea of the bride as an object of family exchange, disregarding her feelings and will. However, the diversity of testimonies and variations between communities shows that these traditions are constantly changing. Thus, critically analyzing warmi urquy not only helps to highlight the tensions between tradition and gender equality, but also promotes community reflection aimed at cultural coexistence with greater respect and recognition of women’s rights.

In the city of Huanta, this traditional practice generates mixed perceptions: while many consider it unfavorable for women, others appreciate it for its role in marital stability and the prevention of divorce. Remaining kneeling for hours in front of those present becomes a symbolic act that seeks to reinforce the importance of family. Although on this occasion the three-pronged whip blessed by a priest was not used, some older women defended its continued use as part of the intergenerational legacy. Despite the changes, the custom remains in different areas of the region and is frequently shared on social media, where it is debated between those who see it as a folkloric vestige and those who consider it a necessary expression of cultural identity. Women continue to be the focus of the ritual, while the groom and his family seek to demonstrate respect and commitment within the ceremony.

One of the limitations encountered in this research reflects the need to deepen our understanding of the ritual from the perspective of the bride’s family, with whom it was not possible to interact due to their own ceremonial commitments and the prolonged wait for the groom’s relatives. Accompanying the bride and her entourage is essential to fully interpret their rituals, perceptions, and preparations, especially considering the predominant use of Quechua and the diplomatic dimension of the event. Similarly, participant observation reduced the scope for conducting further interviews, even with the bride and groom themselves, given their constant involvement in the ceremony. Future research should therefore be expanded to include the perspectives of both spouses, the opinions of the bride’s family, and the events of the following day in their home, where the celebration continues with festivities marking the achievement of “finding” a wife for the son. All of this highlights that the woman remains in a vulnerable position, as she has no close relatives other than the godparents and is exposed to the man’s family.

## Data Availability

The original contributions presented in the study are included in the article/Supplementary material, further inquiries can be directed to the corresponding author.
