# Supplementary material for: “Warmi urquy”: marriage proposals and gender hierarchy in the Peruvian Andes
Source: Front Sociol. 2026 Mar 3;11:1722698. doi: 10.3389/fsoc.2026.1722698 (PMC12994035; doi:10.3389/fsoc.2026.1722698)
Supplement: Supplementary file 1 [file Supplementary_file_1.pdf]

Supplementary Material

1 Supplementary Data

Contact Carlos: In a general comment about machismo with the security guard at the National Autonomous University of Huanta, the conversation arose about the engagement ceremony, the *warmi urquy*, to be carried out in the traditional style. Carlos invited us to take part in the ceremony by posing as the groom's family. He explained that the bride's family had set the condition of holding the engagement in the traditional way.

Carlos: When it had been planned for us to take part as members of the groom's family on an agreed date, and we were at the groom's house door, the mother informed us that, unfortunately, it had been postponed to another date, because the conditions were not in place—especially since the hired musician had not confirmed his attendance.

Groom's mother: She authorized our participation as the groom's family, and we could be present from the beginning of the ritual until whatever time we wished to stay at the ceremony. It was important to pose as family; otherwise, the bride's relatives might have reservations about accepting us during the engagement ritual.

Researchers' visit: The entire ritual lasts through the night, beginning days in advance with the hiring of musicians, godparents, roasted pork, *qarawi* (a Quechua song performed only by women, especially elderly ones), as well as the preparation of *mondongo*, chicken soup, and the provision of food and drinks.

First interview with the couple's family: The groom's father asked us about the purpose of our visit, and we explained it. The mother took us around to see the whole setting and how they had prepared for the occasion. Every visitor who arrived at the house was served *mondongo*, chicken soup, coca leaves, cigarettes, and liquor to help them stay awake.

Interview: With an older attendee, who stated that this is a long-standing tradition where these rituals are practiced, and in this way the family endures over time; because nowadays divorces are frequent. He explained that in the past people never divorced, they maintained family unity. In his own case, it was the same—the engagement ceremony required them to go far from their town, and that is how they have stayed together until today.

Observation: The conversation revolved around the different anecdotes they had experienced during engagement rituals. Some mentioned that they struggled a lot to find a wife because their parents were very demanding in requiring certain rituals to be fulfilled. The younger ones were excited, as it was their first time taking part in the *warmi urquy*.

From time to time, liquor is passed around in plastic containers to avoid breaking glass bottles, since breaking them is considered bad luck. Coca leaves and cigarettes are also shared among family members and visitors to counteract the cold, because the activity begins about an hour before midnight with the trip to the bride's house.

The groom's father and the godparents organize a group with specific roles, such as the person who will knock on the bride's door, and they rehearse what to say (the entire activity is mostly conducted in Quechua). The person chosen to knock must be experienced and someone who has had success in previous engagement ceremonies. He repeats phrases like: *qamunico sumaq rusas waitachayquiman* ("we come to your virgin and beautiful flower"), where "flower" refers to the woman. Great care must be taken to use the word *rusas* ("virgin"), otherwise it would be an offense. If the woman already has children, the questions are asked in a different way. In such cases, there are stories of the bride's father refusing to open the door.

Another role is assigned to the person who discreetly carries the coca leaves, liquor, and cigarettes, and who is expected to be the boldest, since he must serve all the family members gathered at the bride's house. Usually, this is the one who ends up drinking the most liquor. Selecting this person was complicated because no one wanted to accept, so the father, the groom, and the godparents had to persuade him with pleas and small gifts like soda, and the next day, chicken soup.

Other roles include the one who carries the candle, the one in charge of the liquor, the one who brings the roasted pork, toasted corn, and basket of bread, and the one responsible for handling emergencies in case something is missing. Once the entire group is ready, they do a final rehearsal of their duties. They entrust themselves to Mount Razuhuillca, the lagoons, and Almighty God so that everything will go well. Then the procession sets out; in this case, pickup trucks were waiting to take them to the bride's house, which was about half an hour away. Experienced people say that in the past they had to walk for hours to reach the bride's house, but now it is easier, and ancestral customs are changing due to the influence of mass media.

Arrival at the bride's house must be just before midnight. Once we reached the block, each person began to carry out their assigned role, approaching the door quietly and carefully. The conversation was whispered so that only the group could understand. Then, the person assigned to knock, together with the groom's father, began their part—an especially difficult and nerve-wracking task, since they were dealing with an unfamiliar family whose way of life had to be respected, without knowing how they might react.

Interview: With the member of the group assigned to knock on the door. When asked what happens if they don't open, he explained that it is tradition to open before midnight, because otherwise it brings bad luck. He also added that since this was already agreed upon and it was the third *warmi urquy* visit, they were a case with consent, so there would be no major inconvenience.

Observation: As part of the observation, we approached with the groom's father and the person assigned to knock on the door. After several attempts, the door was opened by an elderly man. The groom's father asked softly: *papay qamunicu sumaq rusas waytachaman, warmaywansi riqsinakun, bibliapim nim adanqa manas kammanchu sapallam, chaymi tataytadios maskapum quk waytachata.* On hearing this expression, which was like a plea, the elder (who turned out to be the bride's grandfather) responded: *ajá qamchu kamki chay runa. Haber tapuykamusaq imaninqas familiyay.* He then closed the door and went back inside, making it a condition to first consult what his family had to say.

After a while, the elder returned, saying that only the groom's father and the person responsible for speaking about the *warmi urquy* should enter. We slipped in as well, and the first action was to light a pair of candles as a symbol of respect for the bride's family. Shortly afterward, the electric light was

turned on, and it turned out that the entire bride's family was there, seated in an orderly manner. Upon entering, everyone had to greet all present from the beginning and then find a seat or another spot to endure the cold, since visitors are generally not given much attention (it is common for visitors to "suffer" through this). We sat wherever we could, and no one spoke—only whispers among those sitting next to each other. After a while, the groom's father said: *chiri papay* ("it's cold, father"), to which the grandfather replied *arí* ("yes"), and that was it. Silence returned once again.

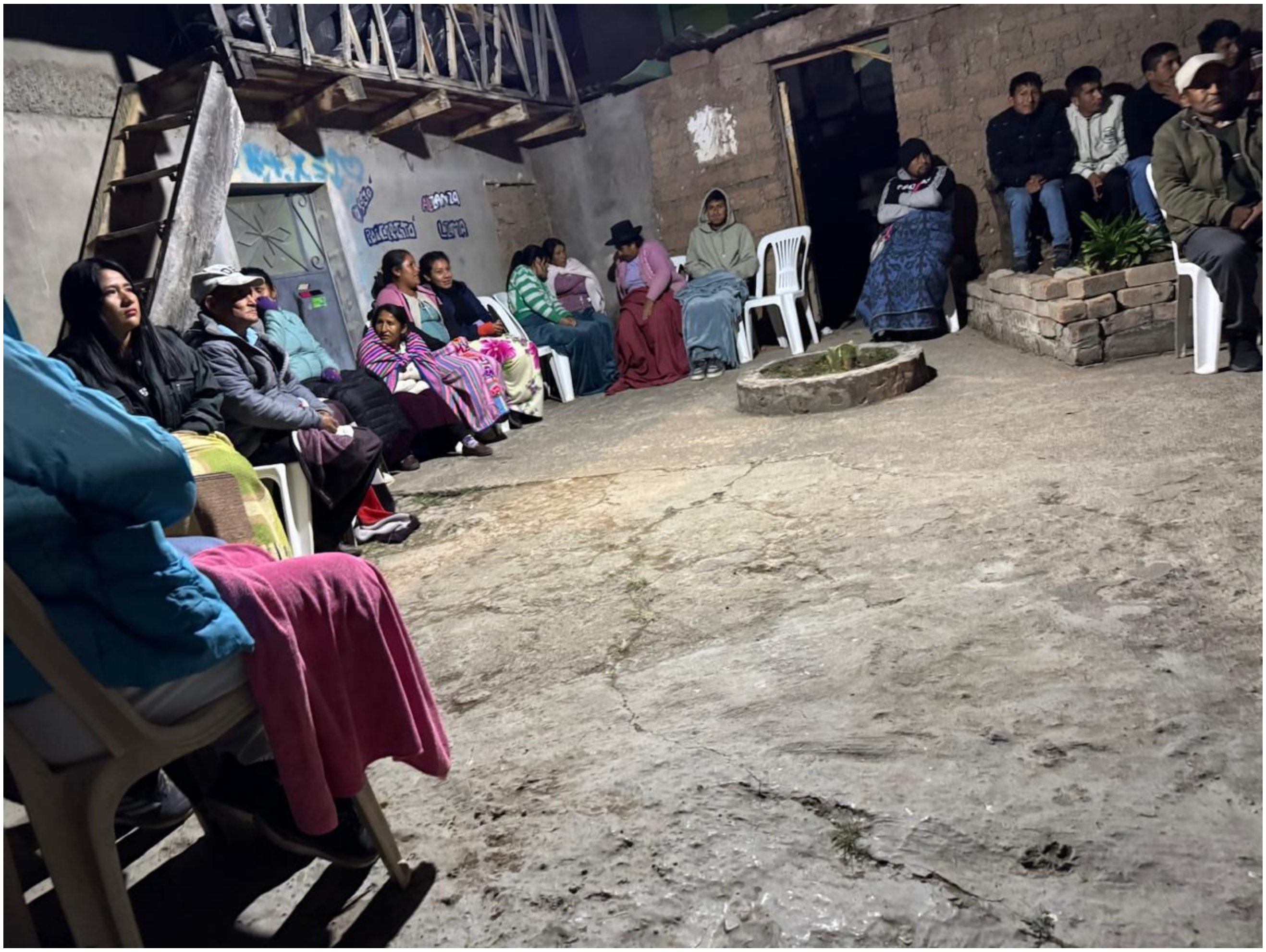

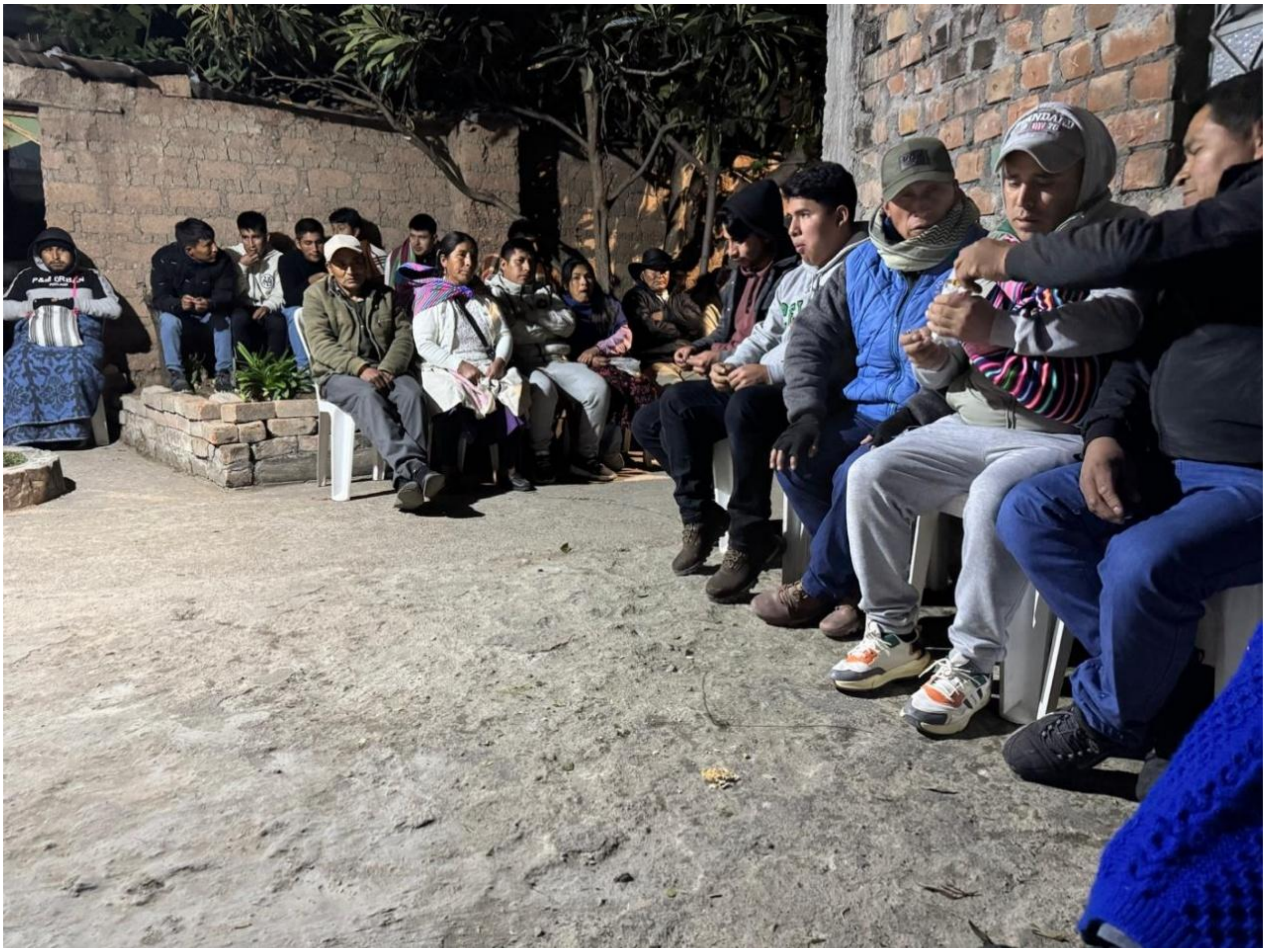

After nearly an hour of silence, with only brief remarks about the cold weather (*chiri*), the groom's father finally ventured to ask if they could serve coca leaves for chewing. The grandfather replied yes. At that point, the service commission stepped in, going around person by person to offer coca leaves and cigarettes.

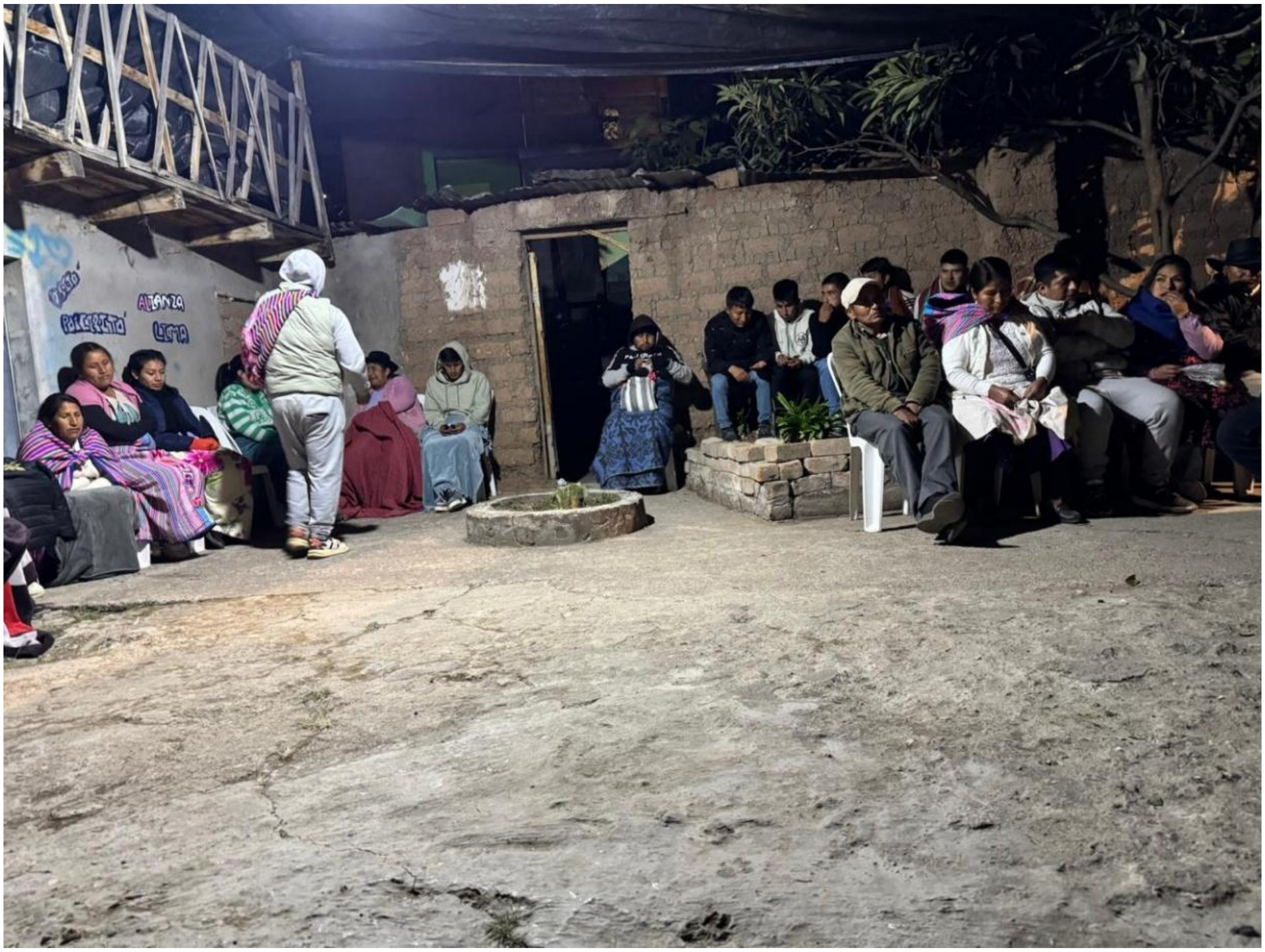
ALIANZA
LIMA

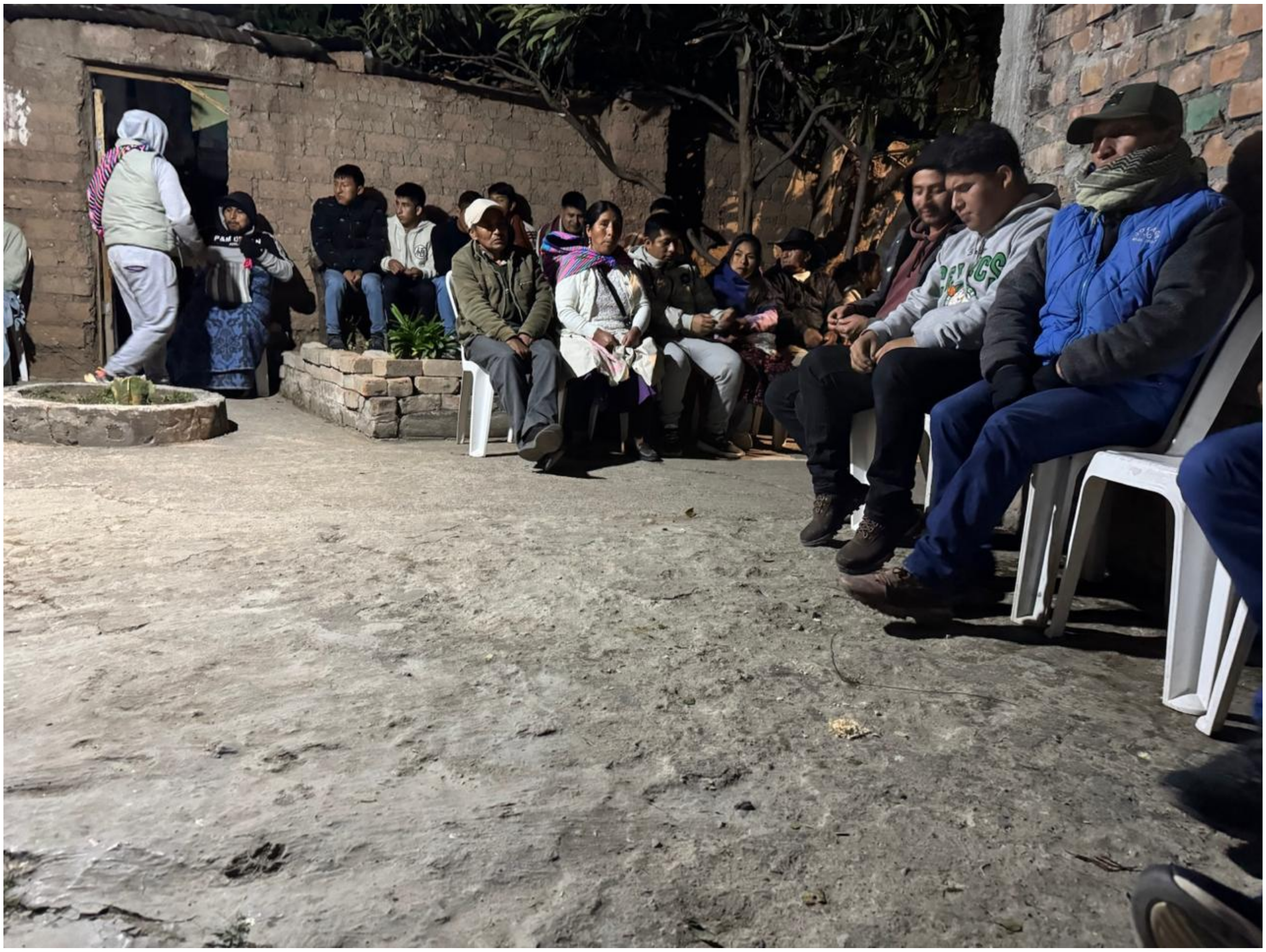

Then came another moment of silence as the cold grew harsher. Once again, the groom's father said *chiri* ("cold"), and two members of the bride's family replied *awa*. This became the cue to ask if they could serve *cañita* (sugarcane liquor). Once more came the answer *arí* ("yes"); then the appointed server began discreetly pouring the liquor. Thus, we spent hours waiting to see what would come next.

An hour passed, and after several rounds of coca leaves, cigarettes, and liquor had been served, the person assigned to knock on the door and lead the ritual finally decided to speak. He said that they had come for a family matter—the son of … wished to start a family. The first thing he did was ask everyone to stand and recite a prayer to Almighty God before beginning to explain the purpose of this well-organized visit.

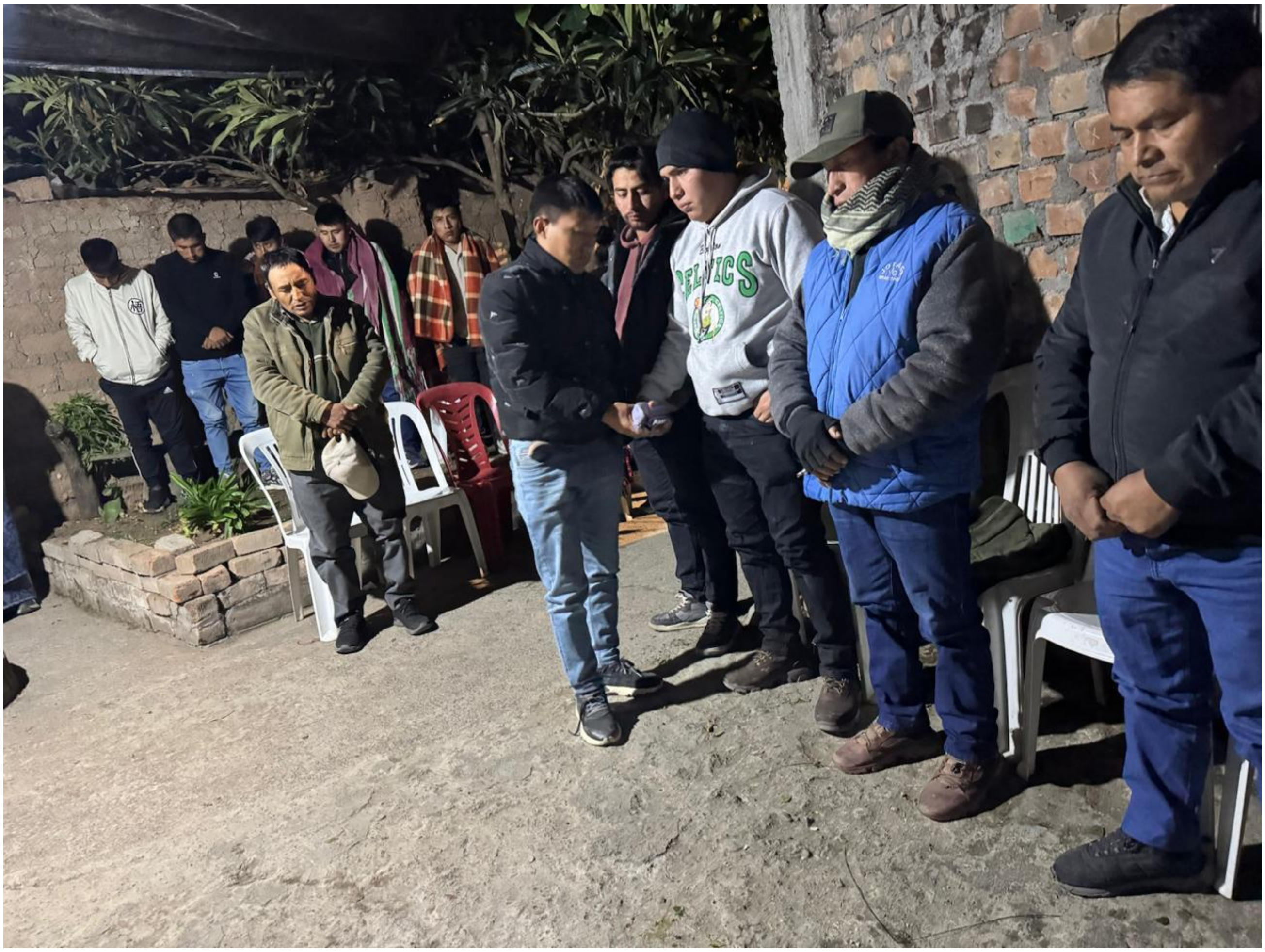

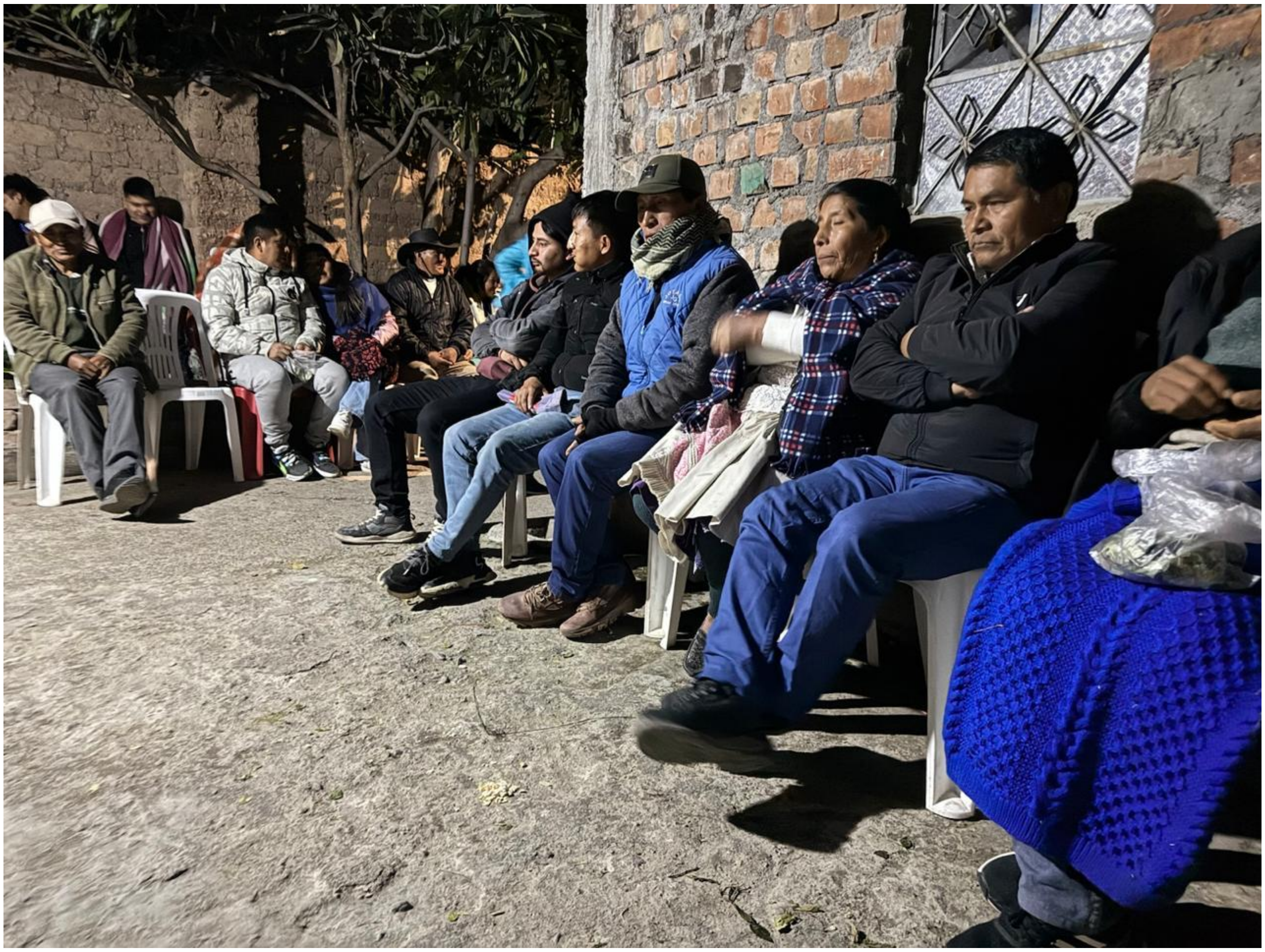

Interview: We asked the woman who was sitting closest to the researchers which one was the bride. She replied that it must be one of them, but that this would take time because there had to be a protocol and the parents had to give their consent for their daughter to leave the house.

Observation: The person in charge of knocking on the door and leading the ritual ceremony hesitated to address the bride's parents, explaining that they had come with good intentions to ask for their daughter's hand. At that moment, the grandfather intervened and said that the father should speak. Then, with considerable fear, the father began to speak, saying that every family had to go through this stage, and he referred to the Bible story of Adam and Eve to begin his speech in Quechua.

Immediately, the bride's father asked the groom's mother to speak, and she decided to talk about her son's intentions, also referring to the Bible. The grandfather thanked the gesture of the visiting parents and then asked the groom to speak. The groom spoke about his intentions and the seriousness with which they were approaching the matter, noting that they had made the request through a ritual in keeping with tradition. Since the bride's parents wished to get to know the groom, he introduced himself, and the bride's family began murmuring about his physical condition, age, background, and expressions. All of this was carried out in Quechua.

When the groom finished his speech, his father intervened, requesting that they also meet the bride. Suddenly, she appeared from a room that had remained closed the entire time. She entered well-dressed, was applauded, and then gave her speech at the request of the groom's father.

Once the introductions were over, they asked the godfather to speak, as he is the most important person in this ritual. He addressed the way both the godmother and godfather should proceed. At that moment, the person assigned to knock on the door said a few words and instructed that the couple sit next to their godparents, who from that moment on would become their family and watch over their future. At the same time, the couple would now regard their godparents as their parents—showing them respect, helping with their tasks, greeting them as mother and father, and obeying their guidance.

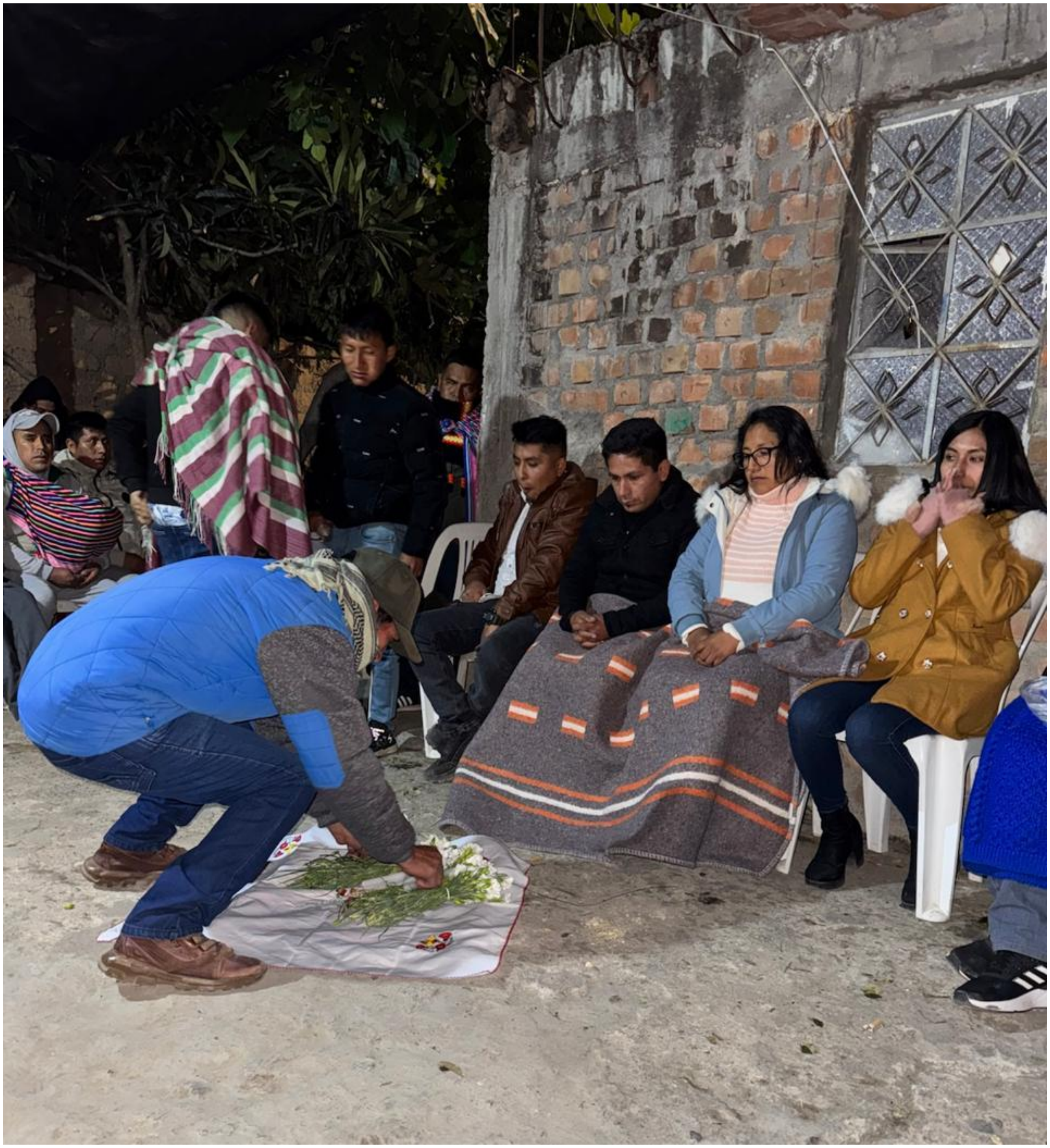

Once the phase of the couple's speeches was finished and they were seated next to their godparents, another scene took place: the person in charge of directing the ceremony requested a white blanket from the materials coordinator. He was given a white cloth with a pair of flowers and two candles,

placed in front of the godparents as prayers were offered to God, all done carefully and respectfully so as not to offend the bride's family.

It was 2:30 a.m., with the piercing cold, cigarettes, coca leaves, and liquor keeping us awake. Then another, even more important and complex ritual began. The presenter asked for a blanket to be brought and placed behind the white cloth with flowers. The couple had to kneel there to receive blessings and advice from almost all of their relatives, both from the bride's and the groom's families, starting with the couple's own relatives.

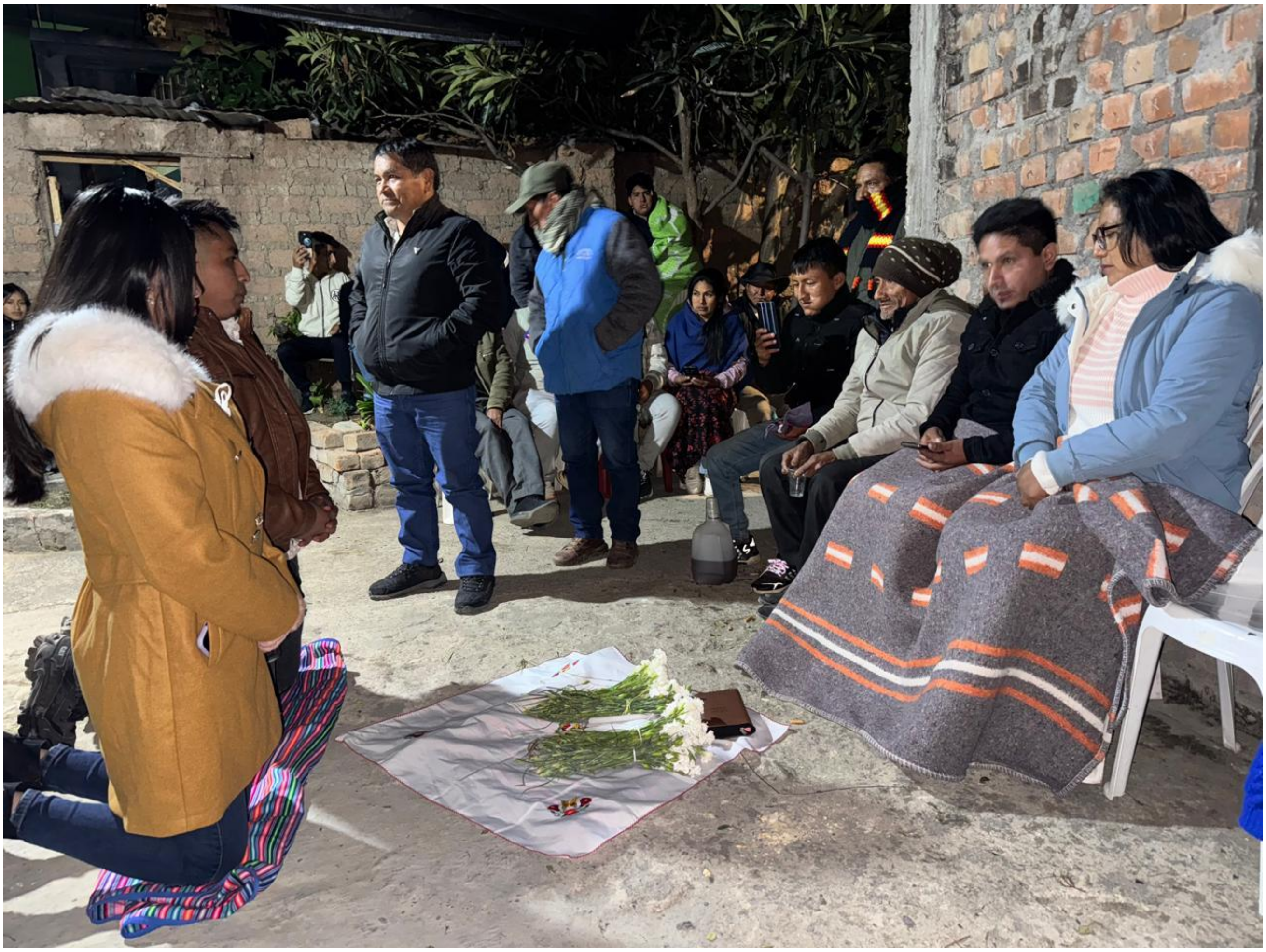

This ritual lasted approximately one hour, during which each family approached the couple and gave advice while they remained kneeling the entire time. Among the many pieces of advice, the woman was told she must know how to cook, wash clothes, care for the children, respect her husband, and help her godparents with their tasks—in essence, recommendations for harmonious living centered on domestic work. Similarly, the man was told that from now on he must leave his friends, give up his single life, and most importantly, avoid using a cell phone, as it is an object that separates families. He must not bring shame to his family through bad behavior and is to entrust his children to the godfather.

The next stage begins with the bride's family. The entire sermon is in Quechua, always starting with the father and then the mother. Many attendees requested the "three lashes" (*chicote*) in the name of God, because they had been given lashes themselves, which were different from what they were

experiencing now. Kneeling is brief for the family members, because they suffered more in their time—and that is how it should be. Each family identifies themselves and begins the sermon, promising to intervene if anything happens, especially in cases of mistreatment by the man. Often, there were warnings directed at the groom to ensure he behaves properly and becomes a respected man. This sermon ritual ends with the couple still kneeling in the cold on a simple blanket, with no right to speak. The mother murmured that her daughter's knees must be hurting and that it should be finished quickly; she was told instead to continue and endure the suffering, as that is part of having a husband.

Once the sermon from the parents and close relatives ended, the next stage began with the godparents, who held a pair of rosaries in what is called the "Rosary exchange." Each rosary was placed around the couple's necks, followed by a symbolic exchange with prayers among the godparents. Then they were asked to stand and light the candle along with the flower that had remained on the white blanket the entire time.

.

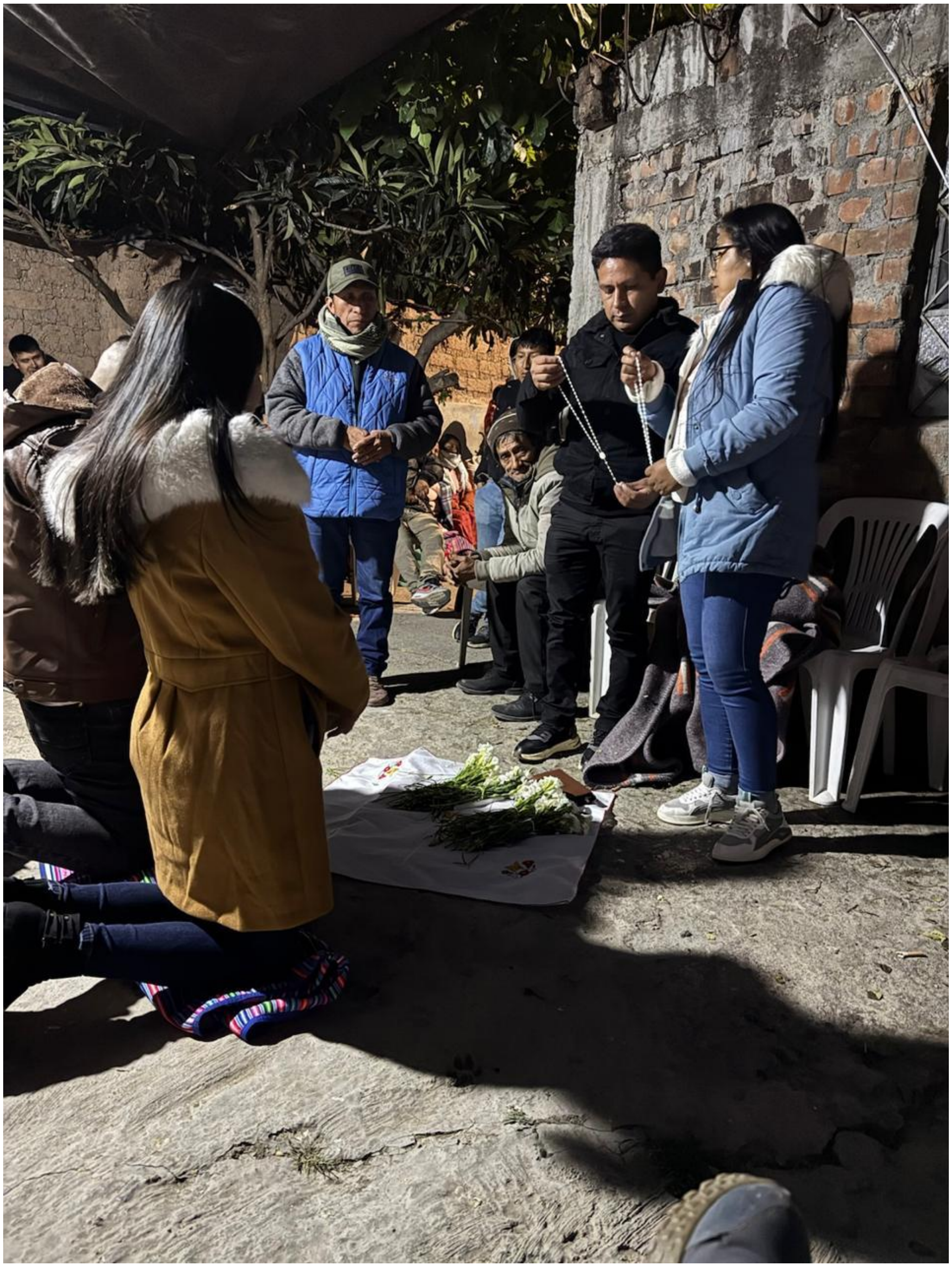

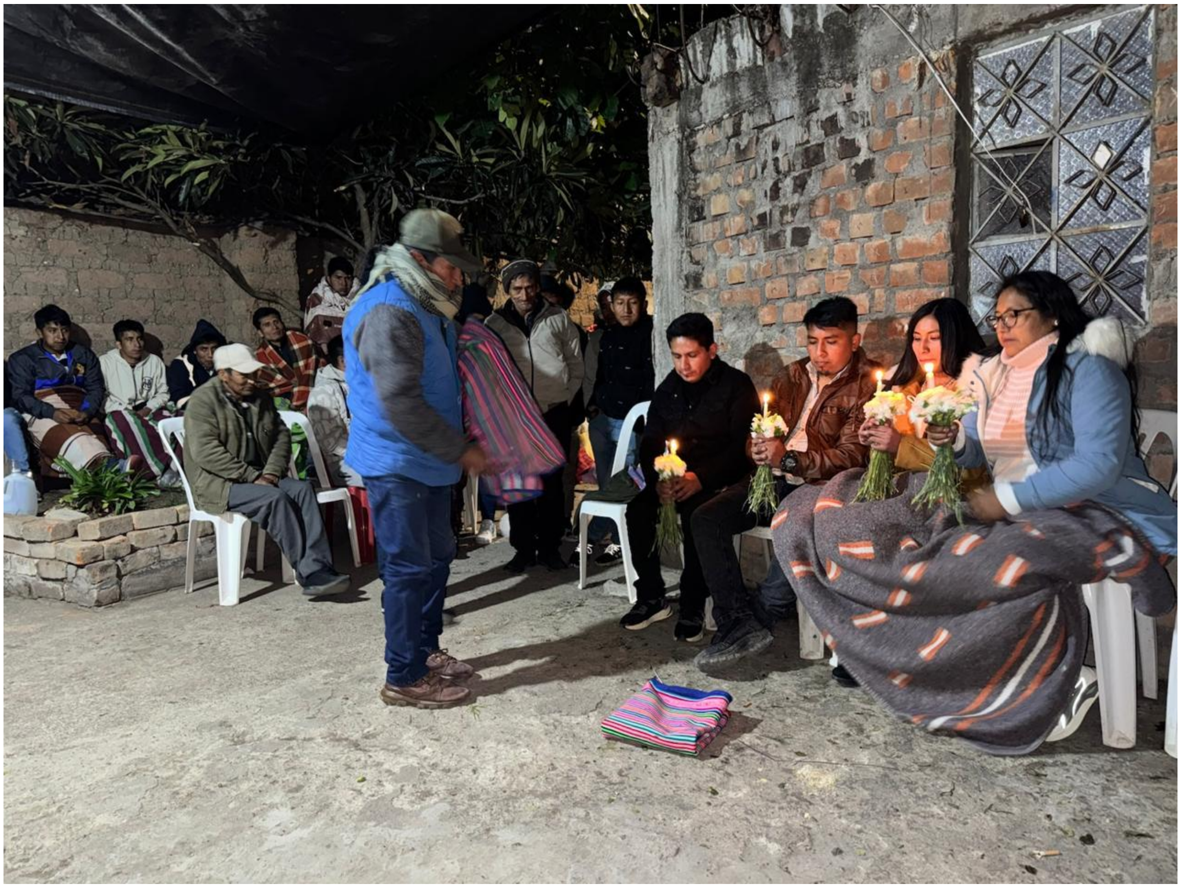

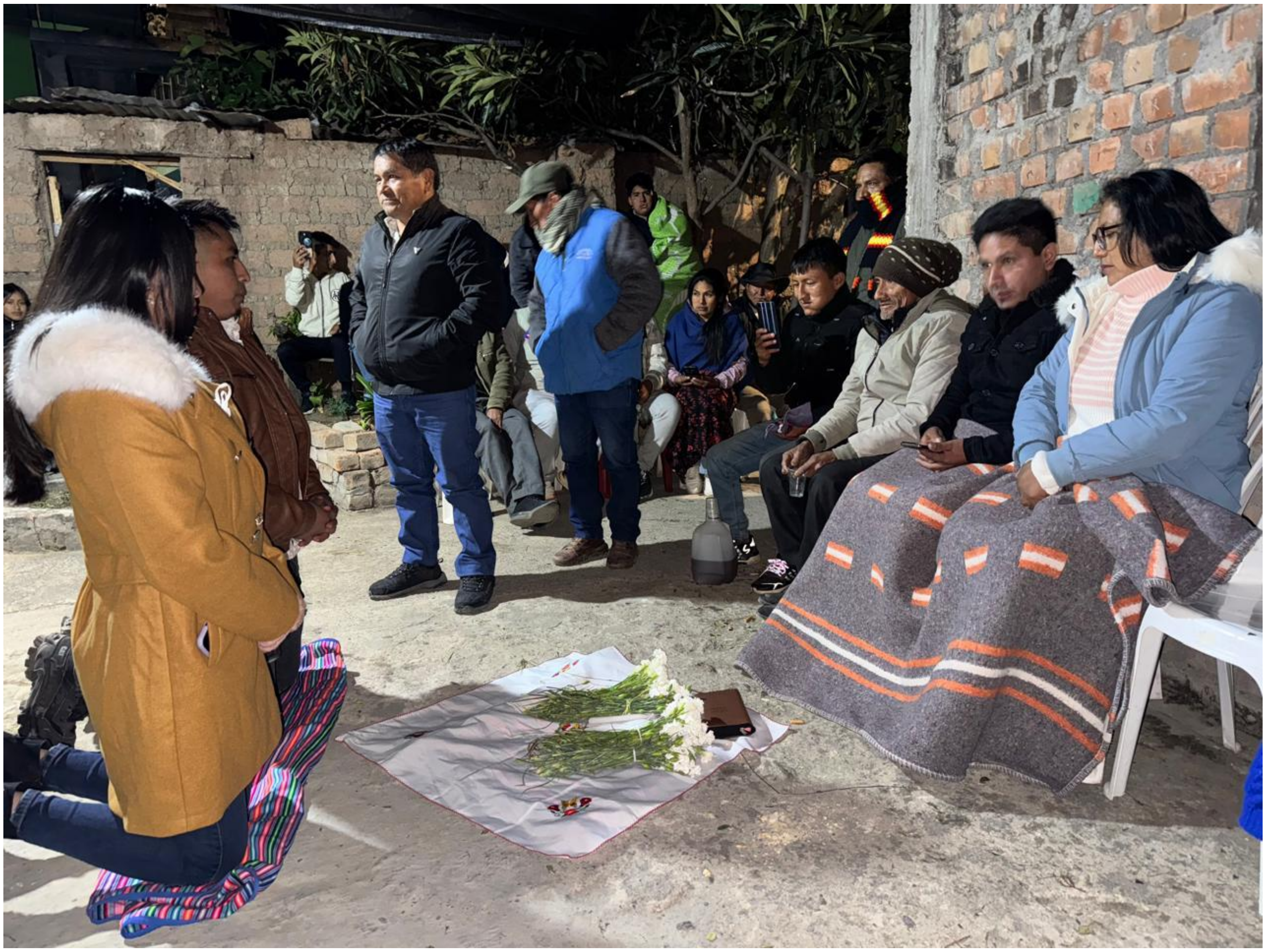

Once the Rosary exchange and the symbolic presentation of a commitment ring to the bride were completed, the couple sat next to their godparents, and another stage of the ritual began. The person in charge of knocking on the door requested that the materials commission bring a blanket and a white cloth, which was then spread out in front of the bride's parents.

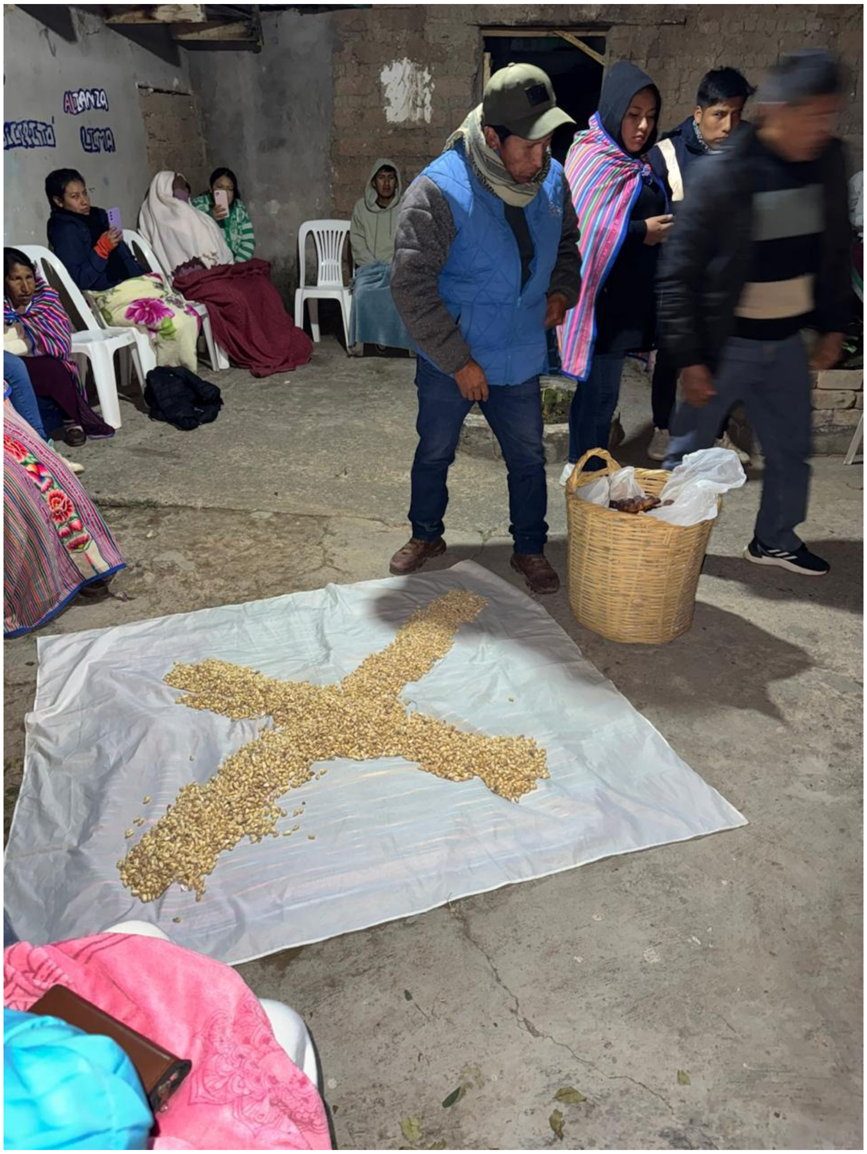

The person in charge placed the blanket and white cloth in front of the bride’s parents and decided to spread toasted corn (cancha, qamka) in the shape of a cross. At the same time, he asked another materials commission to bring the remaining items, including: baby-shaped bread (wawa), Inca Kola soda, a pair of bottles of sugarcane liquor, two boxes of beer, packaged rolls, and finally, roasted pork.

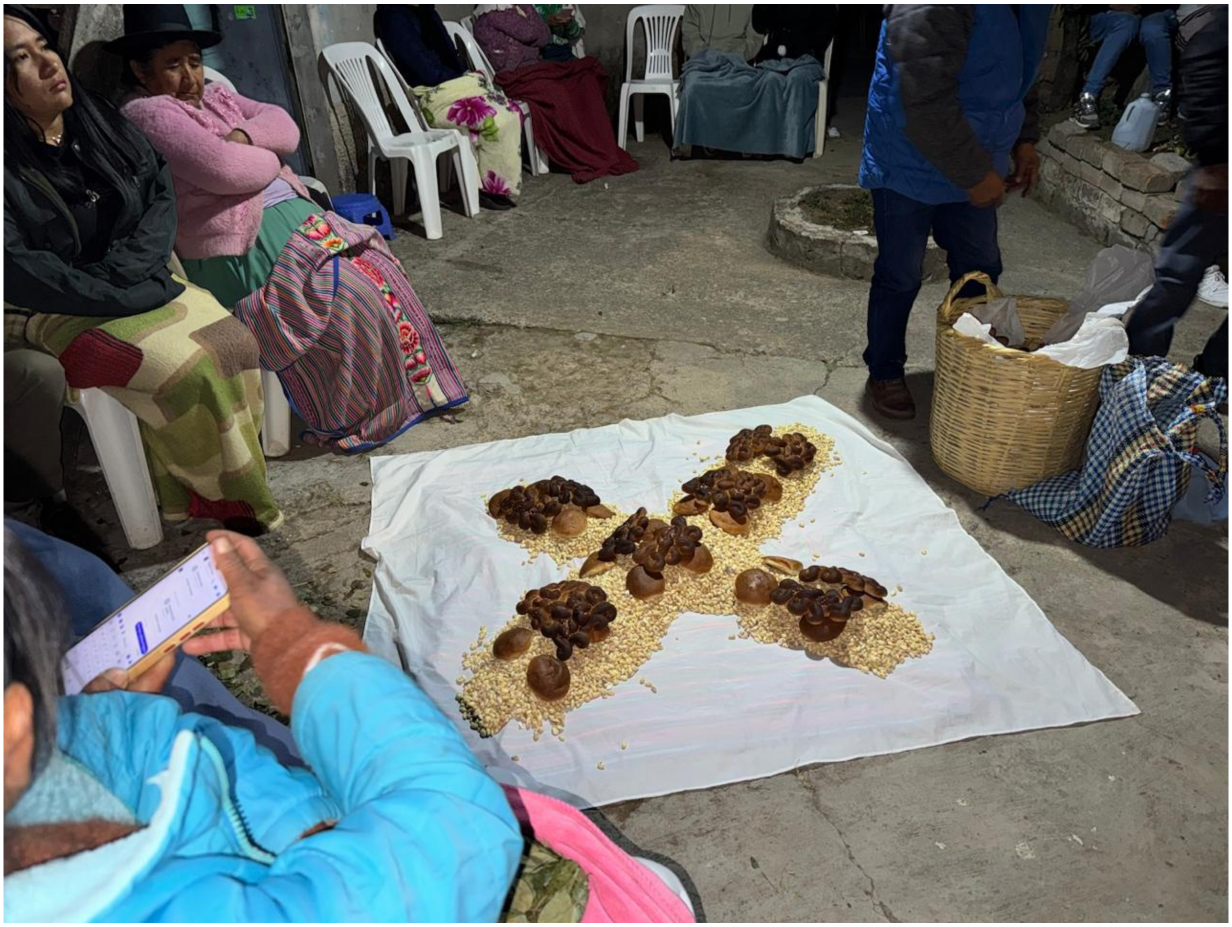

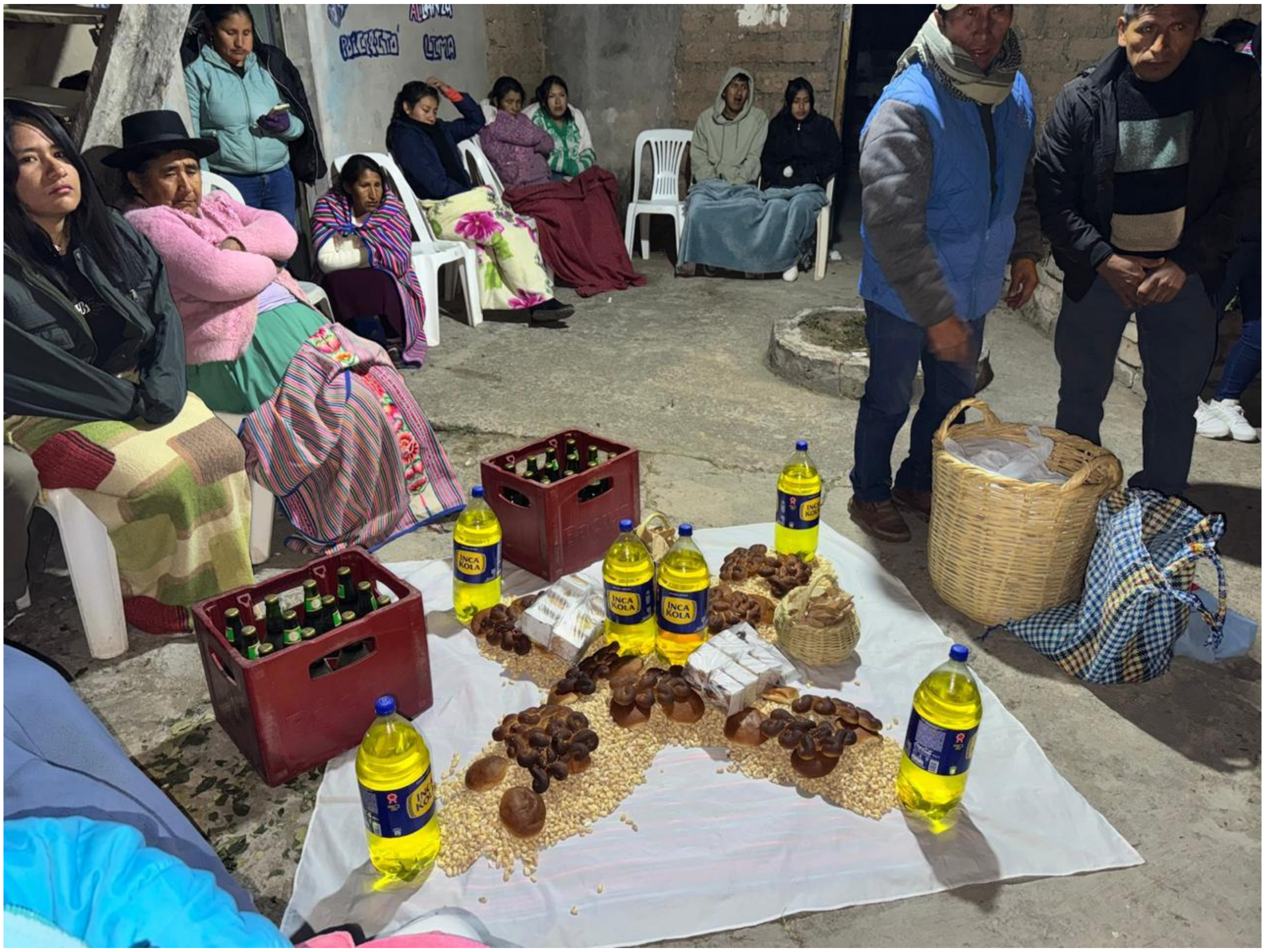
INCA KOLA

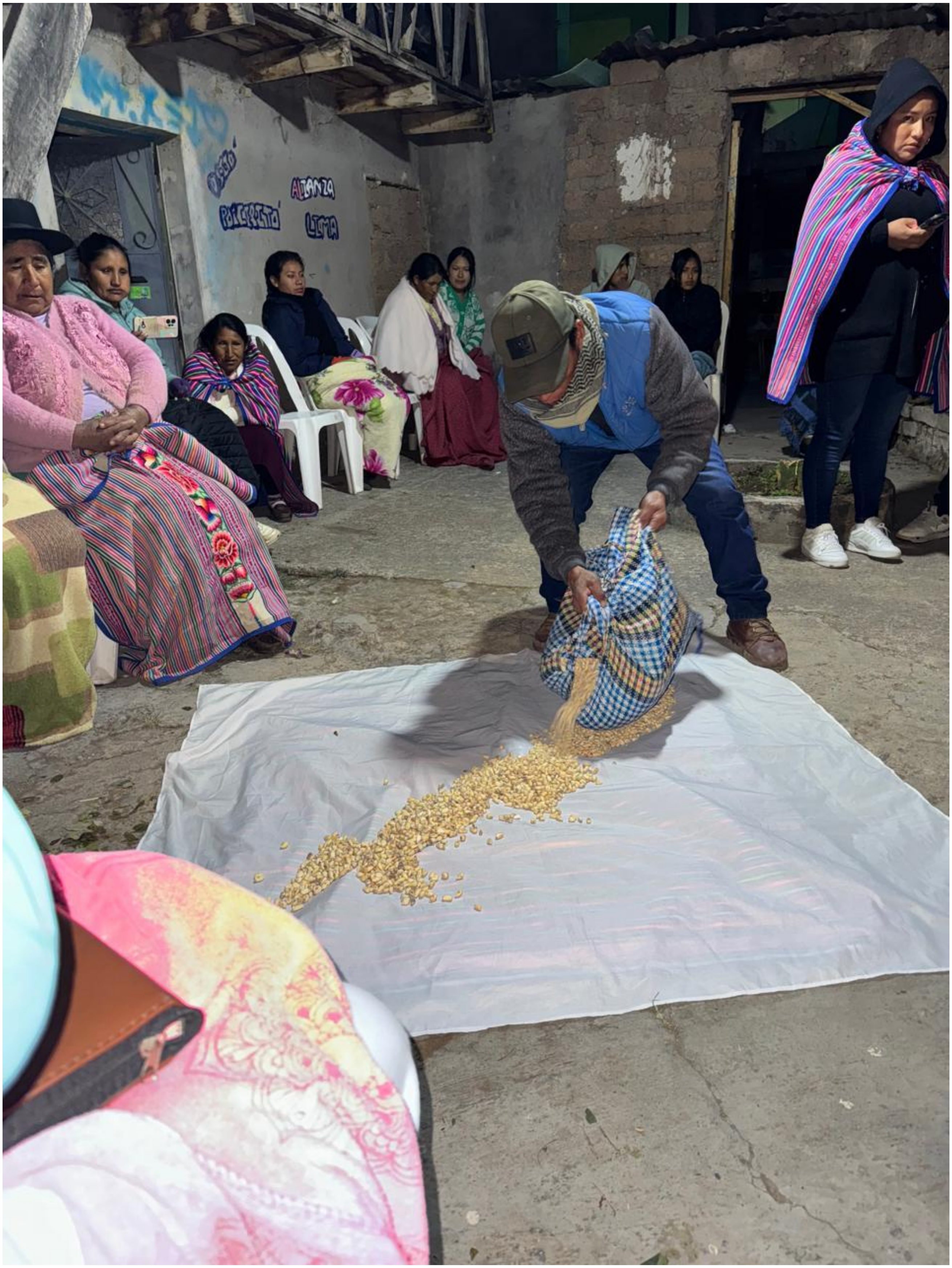
ALIANZA
LIMA

Once the offering was completed—which many whispered symbolized the “exchange of the bride”—the person in charge decided to ask everyone present to participate in a prayer.

It was already 2 a.m., the cold had intensified, and an atmosphere of trust began to develop through the exchanges and the words spoken by each attendee. At that moment, the grandfather intervened, realizing he had forgotten something important: when the wedding date should be set, before all the offerings were collected. The godfather and the bride’s father decided to intervene very carefully so as not to upset the grandfather’s emotions. They decided that the groom would speak with the godfather, and finally they agreed on a date two years later, which did not please the grandfather.

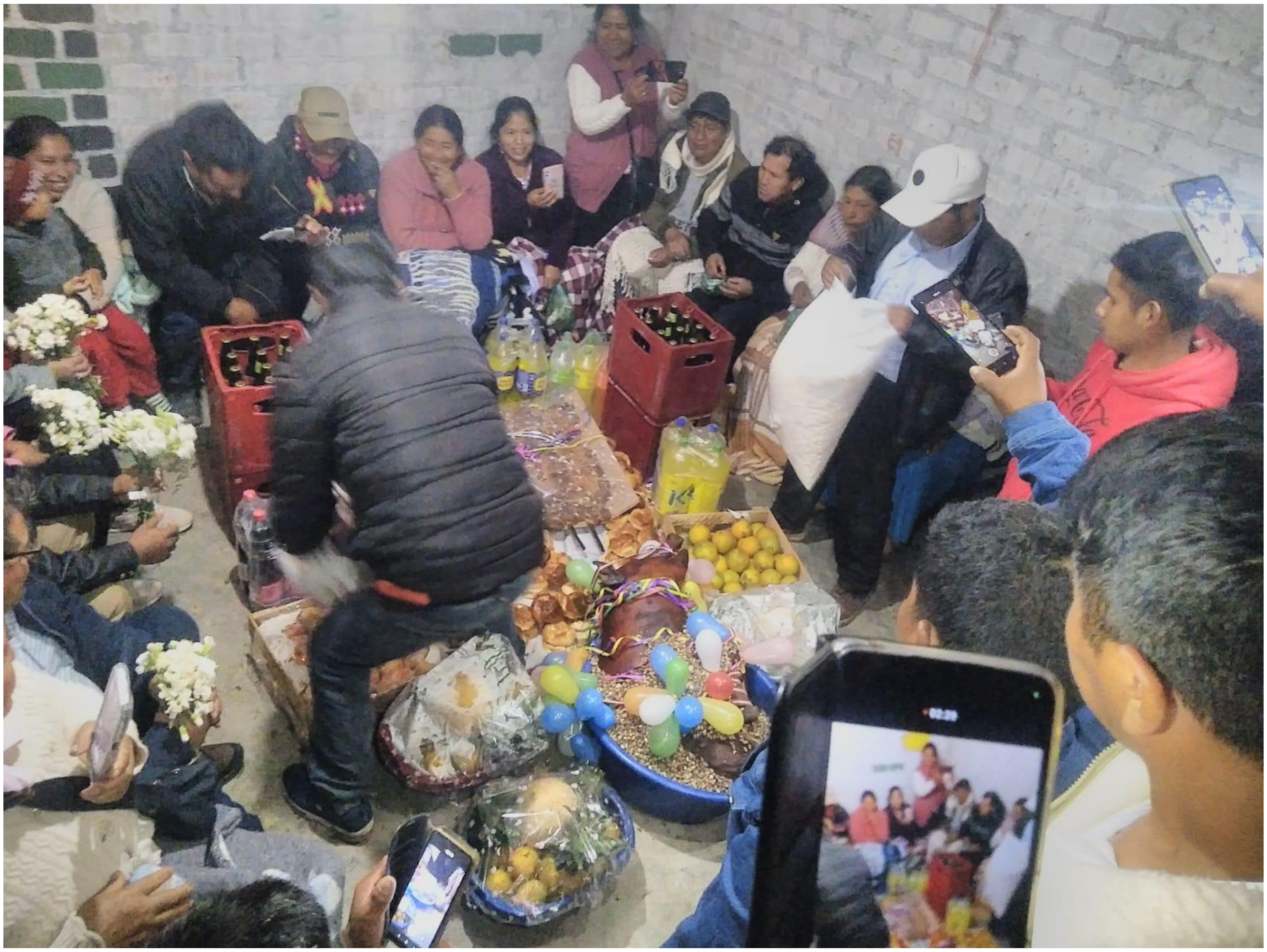
Another setting and another *hand-asking* ceremony in the same province, with similar characteristics, in which roast pork is always present. Image updated on January 5, 2026; the fieldwork corresponds to October 2025 and documents another *hand-asking* ceremony.

.

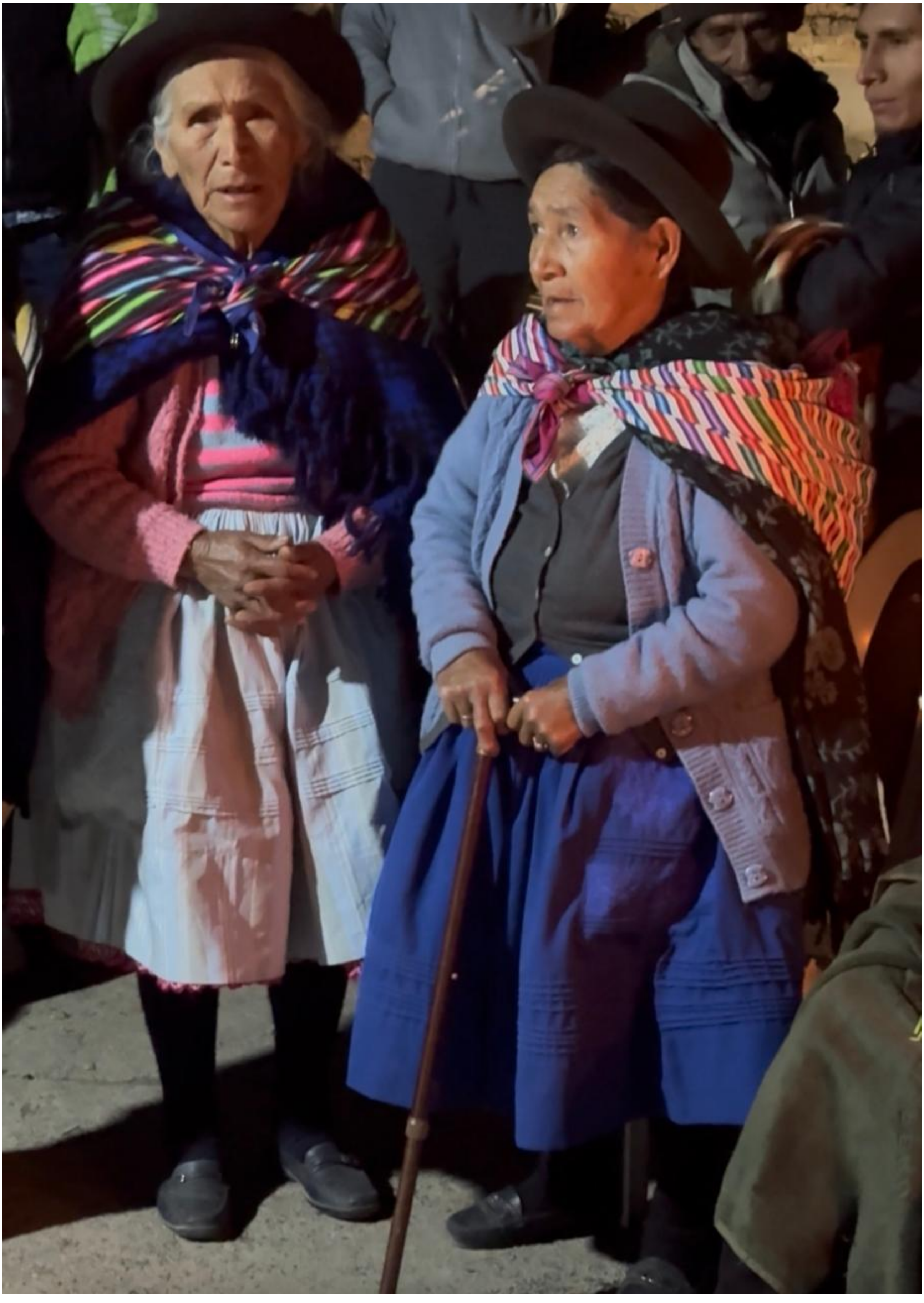

At that moment, the groom's father asked for permission to bring in the musicians, which was granted. The celebration then began with harp and violin music and singing. Liquor was consumed, and a dance ensued involving all the families.

VIDEO (several)

Observation: By around 4 a.m., unusual activity began—it was time to leave and take the bride. The bride's family decided to continue drinking, perhaps as a way of showing affection. The dancing and drinking went on. The surprise was that the service commissioner was completely drunk because everyone had been toasting with him.

They then decided to leave the house. It was customary to leave a relative of the groom behind to continue drinking, so that the next day they would come to collect him with music and liquor.

Once they left, a kind of confrontation occurred, as if mocking the bride's family, pronouncing words indistinguishable on the audio. They departed in the early morning, around 5 a.m., heading to the groom's house in the trucks waiting at the door. Upon arriving at the groom's house, chicken soup and mondongo prepared for the occasion were served.

This marked the beginning of the main celebration at the groom's house.

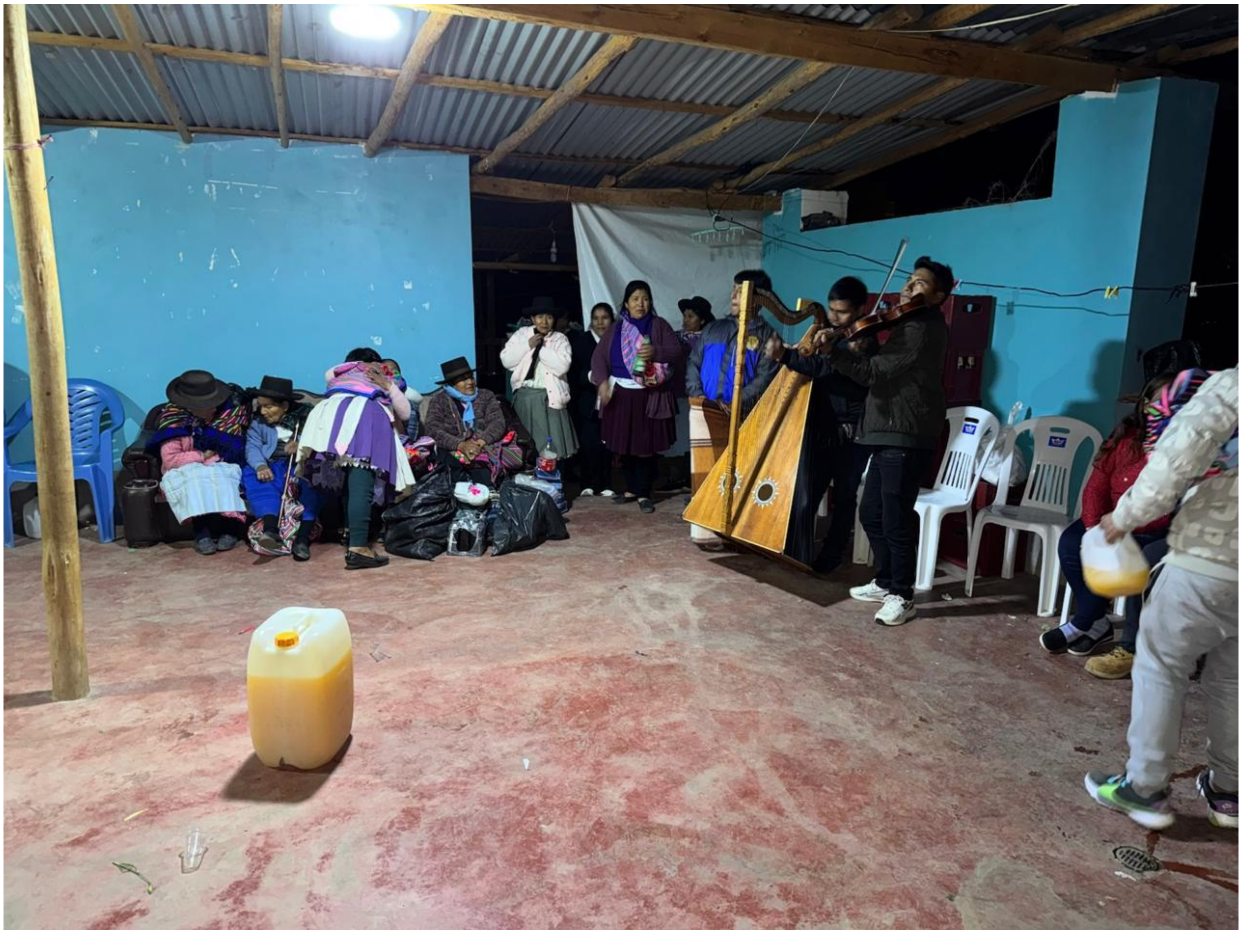

When asked about the total expenses, they estimated it was around 10,000 soles, not including the older, more traditional practices of the past. They noted that the town's traditions are changing due to modern influences.
